# Rapidly adaptable automated interpretation of point-of-care COVID-19 diagnostics

**DOI:** 10.1038/s43856-023-00312-x

**Published:** 2023-06-23

**Authors:** Siddarth Arumugam, Jiawei Ma, Uzay Macar, Guangxing Han, Kathrine McAulay, Darrell Ingram, Alex Ying, Harshit Harpaldas Chellani, Terry Chern, Kenta Reilly, David A. M. Colburn, Robert Stanciu, Craig Duffy, Ashley Williams, Thomas Grys, Shih-Fu Chang, Samuel K. Sia

**Affiliations:** 1 Department of Biomedical Engineering, Columbia University, New York, NY 10027 USA; 2 Department of Computer Science, Columbia University, New York, NY 10027 USA; 3 Department of Electrical Engineering, Columbia University, New York, NY 10027 USA; 4 Department of Laboratory Medicine and Pathology, Mayo Clinic, Phoenix, AZ 85054 USA; 5 Safe Health Systems, Inc., Los Angeles, CA 90036 USA

**Keywords:** Biotechnology, Biological techniques, Signs and symptoms, Diagnosis, Epidemiology

## Abstract

**Background:**

Point-of-care diagnostic devices, such as lateral-flow assays, are becoming widely used by the public. However, efforts to ensure correct assay operation and result interpretation rely on hardware that cannot be easily scaled or image processing approaches requiring large training datasets, necessitating large numbers of tests and expert labeling with validated specimens for every new test kit format.

**Methods:**

We developed a software architecture called AutoAdapt POC that integrates automated membrane extraction, self-supervised learning, and few-shot learning to automate the interpretation of POC diagnostic tests using smartphone cameras in a scalable manner. A base model pre-trained on a single LFA kit is adapted to five different COVID-19 tests (three antigen, two antibody) using just 20 labeled images.

**Results:**

Here we show AutoAdapt POC to yield 99% to 100% accuracy over 726 tests (350 positive, 376 negative). In a COVID-19 drive-through study with 74 untrained users self-testing, 98% found image collection easy, and the rapidly adapted models achieved classification accuracies of 100% on both COVID-19 antigen and antibody test kits. Compared with traditional visual interpretation on 105 test kit results, the algorithm correctly identified 100% of images; without a false negative as interpreted by experts. Finally, compared to a traditional convolutional neural network trained on an HIV test kit, the algorithm showed high accuracy while requiring only 1/50th of the training images.

**Conclusions:**

The study demonstrates how rapid domain adaptation in machine learning can provide quality assurance, linkage to care, and public health tracking for untrained users across diverse POC diagnostic tests.

## Plain language summary

It can be difficult to correctly interpret the results of rapid diagnostic tests that give a visual readout, such as COVID rapid tests. We developed a computational algorithm to interpret rapid test results using an image taken by a smartphone camera. This algorithm can easily be adapted for use on results from different test kits. The algorithm was accurate at interpreting results obtained by members of the public using various COVID rapid tests and diagnostic tests with similar outputs used for other infections. The use of this algorithm should enable accurate interpretation of rapid diagnostic tests by members of the public and hence enable improved medical care.

## Abstract (web summary)

Arumugam, Ma et al. automate the interpretation of lateral flow assays by applying their software architecture to photos taken with a smartphone camera. The approach is rapidly adapted to other test kits using only a fraction of the images required for existing methods, with all test results successfully classified.

## Introduction

A large and diverse number of POC diagnostics, including lateral-flow assays (LFAs), are becoming widely used by the public and endorsed by policymakers, but to ensure patient safety and enable public health monitoring, assurance of correct assay operation and interpretation of results—two standardized quality-controlled processes for diagnostic tests in a centralized laboratory—are critical but not currently performed in decentralized settings. Specifically, errors in these steps hinder their deployment in primary care clinics and homes^1–5^ in challenging cases, especially those involving assays with absent control bands, failure to recognize faint bands, or inability to identify control vs. test bands^6,7^. With the introduction of SARS-CoV-2 antigen LFAs, these errors with test interpretation have been confirmed with community health workers^8^, with considerable uncertainties about how to perform and interpret different rapid tests reported widely by the general public^9,10^, and the FDA requiring rapid test makers to “facilitate results reporting by both the healthcare provider and the individual”^11,12^. A recent study demonstrated low interpretation accuracies with untrained workers necessitating modified instruction guides^13^. With the popularity of LFAs rising among the public^14,15^, user errors that jeopardize interpretation of results, as well as incorrect and unreliable public health monitoring (e.g., based on self-reporting of results^16–19^), could become increasingly widespread in the coming age of digital health^20–23^.

Mobile apps that automatically interpret results from point-of-care diagnostic tests present an opportunity to address these challenges^2,24–26^, with high acceptability among healthcare workers^27^ for HIV and sexually transmitted infections^28,29^, but current approaches are not scalable. Specifically, current approaches require either images to be collected by custom smartphone hardware attachments^5,30–38^, complex custom calibrations^39,40^, or are designed to work retrospectively with a large library of pre-collected images^41,42^/expert-labeled training images ranging in the hundreds^28,29,43–45^ to thousands^8,46^ specific to a test kit format (Supplementary Table 1 and Supplementary Table 2 show side-by-side comparisons of different ML approaches for analyzing diagnostic images and smartphone-based diagnostic tests, respectively), including SARS-CoV-2 rapid tests^46^. Without an adequate number of images for training, these algorithms may fail to accurately interpret the signal from test kits due to the large domain gap caused by different kit designs and environmental factors (e.g., lighting and angle of image capture). These requirements necessitate, in the midst of a fast-moving pandemic, the procurement of large numbers of rapid tests and validated clinical specimens and the availability of experts for labeling, for every new test kit format.

**Fig. 1:**
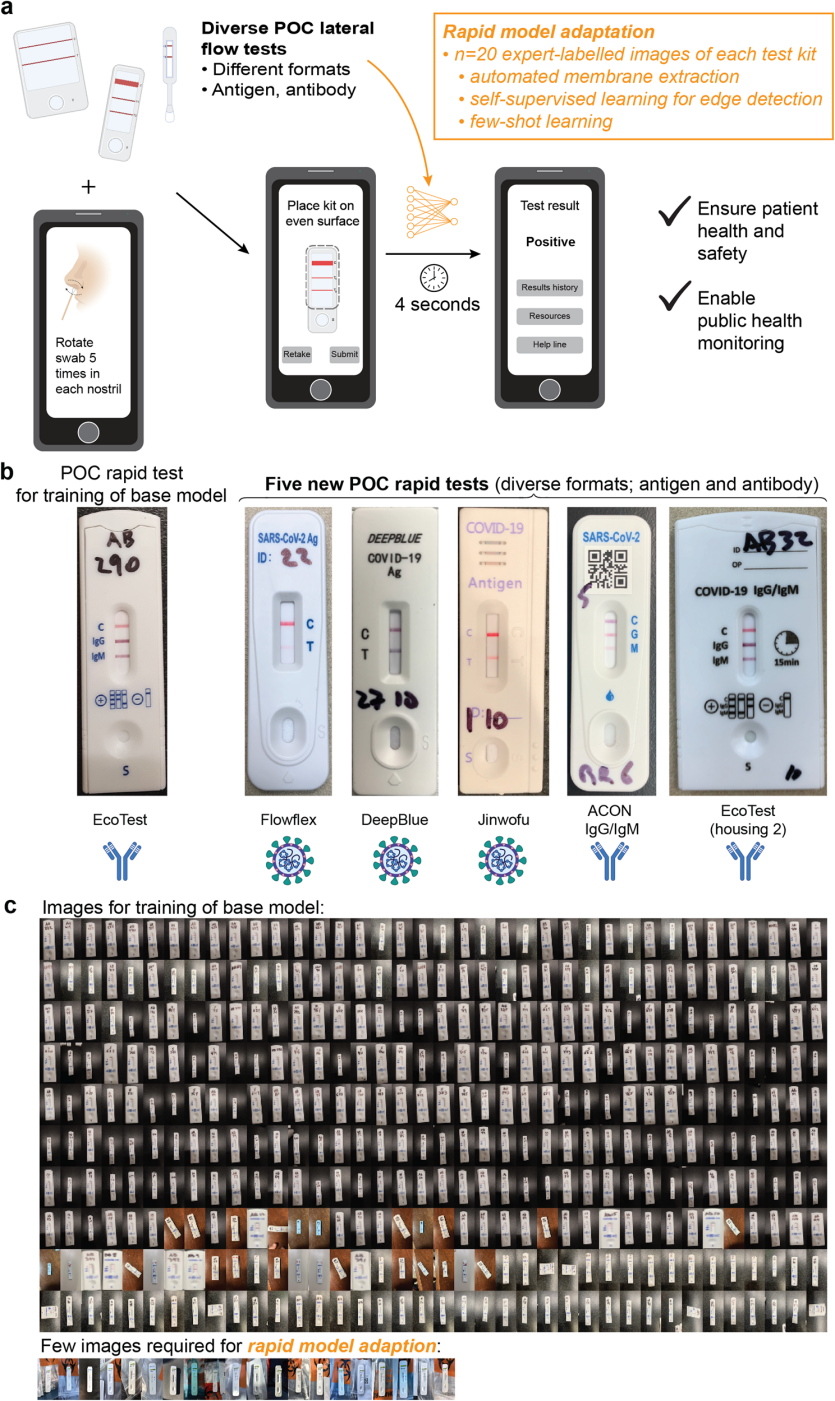
Overall approach of a rapidly adaptable model for interpreting images of rapid tests that require few training images. **a** Overall process of automatically interpreting images from a diverse set of LFAs that span analytical targets, number of test and control bands, and housing and form factor. From a raw image of a LFA, a smartphone can automatically and accurately interpret the result within seconds, using a pre-trained machine-learning model that has been adapted to a specific test kit requiring only 20 images of each new rapid test kit. The considerable reduction in training images can bypass the procurement of large numbers of different types of rapid test kits and expert labeling with thousands of validated specimens per test kit, which is challenging during a pandemic, while ensuring patient health and safety and enabling public health monitoring of results. **b** Images of a base LFA kit (EcoTest) for pre-training the model, and five new COVID-19 LFA kits (including both antigen and antibody tests) that are interpreted using a rapidly adapted model. **c** Actual images used for training of a base model, and for rapid model adaptation for a specific new LFA test kit.

Here, we demonstrate an approach that can rapidly adapt to interpreting new POC diagnostic tests (Fig. 1a), such that large, diverse, and dynamic sets of rapid tests can be interpreted accurately without extensive procurement of specific test kits, validated specimens, and experts for labeling. This approach, called AutoAdapt POC, achieves the goal of rapid adaptation with the development of three components, automated membrane extraction, self-supervised learning specifically designed to preserve image edges that are critical for recognizing faint test kit images, and few-shot learning to adapt a pre-trained model to different test kits. Few-shot learning has been employed in applications ranging from computer vision to robotics^47–49^ to adapt a classifier originally trained on data from different domains^50–54^ with only a few labeled images in the new target domain. (Domain adaptation^55,56^ using adversarial learning^57^ still requires a large number of unlabeled images from the target domain.) In AutoAdapt POC, we exploit the domain-invariant patterns within the edge area of bands. We optimize the feature extraction module so that the extracted feature can be used to reconstruct the edge image with minimal deviation from the original edge image. Preserving the edge patterns in an image is important since distinct edge patterns carry unique attributes. With our feature extractor made sensitive to edge patterns, it can recognize faint test kit images and be robust to the domain gap, removing the need for complex pre-processing calibration (such as white balance or shadow removal). Since the edge-filtered image can be produced by automatic edge detection tools, they can be used to train the model in a self-supervised way without needing a time-consuming process to collect manual labels. Note that compared to conventional approaches that use edge detectors (e.g., Sobel^58^, SIFT^39,59^, or canny edge detector^40,60^) to directly extract features, our approach uses edge detection to generate the label for the self-supervision task to enforce the knowledge of edge sensitivity into the feature extractor.

To adapt to a new POC kit, zone areas from 10 to 20 images of a new assay kit are automatically cropped, and the pre-trained model, i.e., base model, adjusts its weights using supervised contrastive learning over a mixture of data from the original test kit and a small set of new data from the target test kit. The automated and modular analysis of bands (instead of entire test kits), along with encoding and decoding of edge-filtered images for self-supervised learning, are crucial to the universality and accuracy of the approach. We pre-trained a base model using expert-labeled images from one test kit (i.e., base kit) and adapted the model to five other commercial COVID-19 LFAs (detecting either antigen or antibody, Fig. 1b) and a non-COVID-19 LFA kit (i.e., HIV test kit). Compared to traditional methods, only a fraction of training images is required for each new test kit (actual images shown in Fig. 1c). The approach was further validated with untrained users in a drive-through tent for COVID-19 testing, with the algorithm correctly identifying 100% of antigen and antibody tests. In comparison to visual interpretation by experts, the algorithm correctly identified 100% of COVID-19 antigen test kits run at specified virus titers (low, medium, and high). AutoAdapt POC provides quality assurance, linkage to care, and public health tracking to untrained users operating a diverse and dynamic set of POC diagnostic tests.

## Methods

In “Model architecture”, we provide an overview of the architecture for the AutoAdapt POC model, followed by a detailed explanation of each module. In “Data acquisition”, we overview methods to collect the images used to train the base model as well as adapted models for five additional COVID-19 test kits. In “Model evaluation”, we overview methods for evaluating the model’s performance on (1) COVID-19 drive-through study with untrained users, (2) comparative assessment with contrived samples, and (3) HIV rapid test kits.

### Model architecture

#### Overview of pipeline

**Fig. 2:**
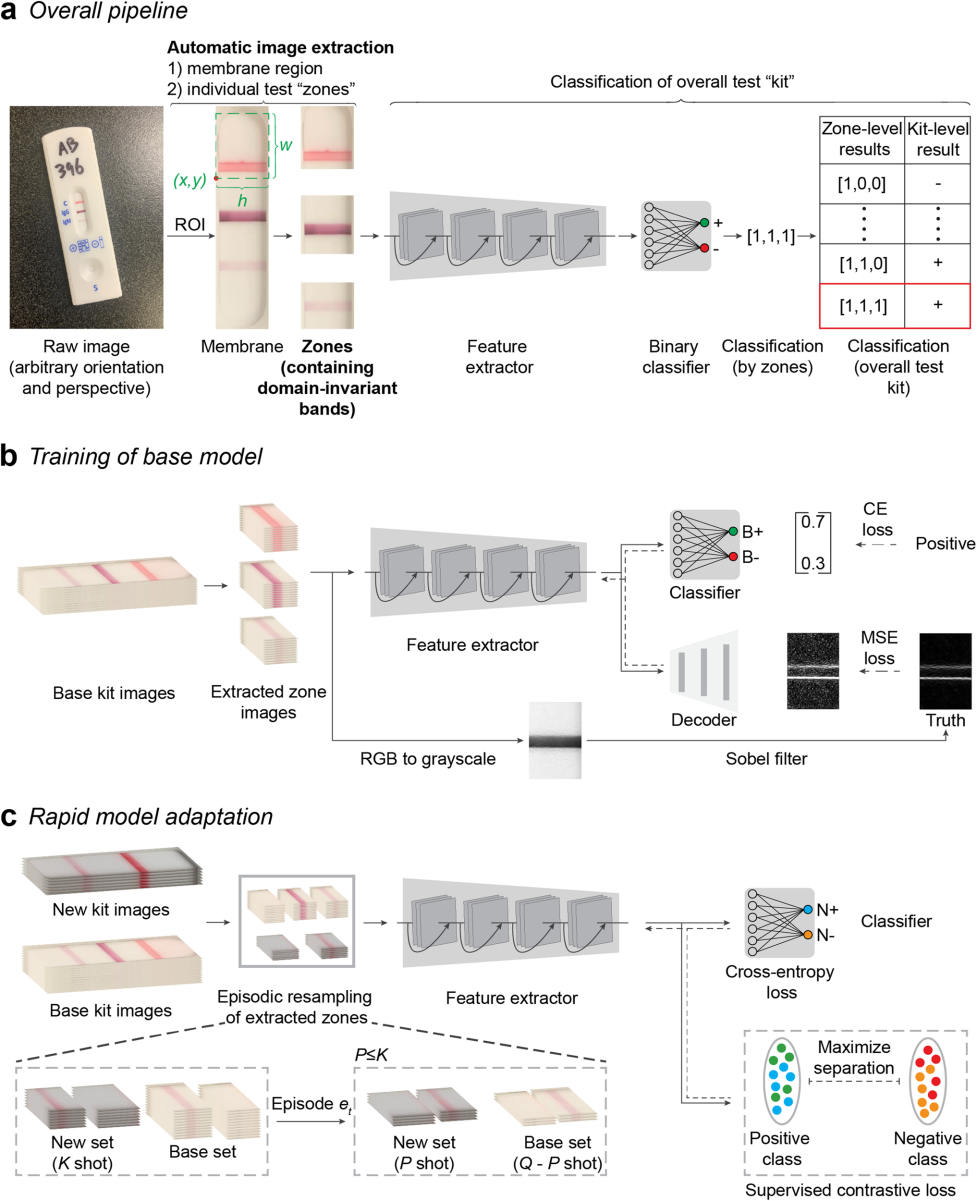
Overview of AutoAdapt POC machine-learning pipeline. **a** From a raw input image of an assay kit, a correction of orientation and perspective is applied to segment an image of an assay kit. From the assay kit image, a segmentation model based on Mask R-CNN is used to extract the membrane region of interest (RoI). Based on measured kit-specific parameters (details in Supplementary Table 3), individual zones are cropped, and passed through a software pipeline consisting of a feature extractor followed by a binary classifier. Classification of each zone allows, via a kit-specific lookup table, for a final classification of assay result (kit-level classification or result) as positive, negative, or invalid. **b** The feature extractor is pre-trained on the base kit using self-supervised learning task over edge-filtered patterns and fully-supervised binary classification task. For each zone, fully-supervised binary classification is carried out with cross-entropy loss with the annotated binary labels. Sobel filter is used to highlight the edge pixels between the band and the background of the membrane. The edge image after normalization is used as ground truth and the learning process is used to reconstruct an image that resembles the ground truth edge image, with the quality measured in MSE (Mean Square Error). The solid and dashed arrows indicate forward processing and gradient backpropagation, respectively, during the learning process. **c** Model adaptation is carried out by supervised contrastive learning to regularize the feature extractor and fully-supervised learning to learn an adapted classifier for the new kit. A sampling strategy to build an episode with Q (e.g., 32) images per class is used: for each class (positive or negative), given K (e.g., 10) images available, P (e.g., 4) images are subsampled from the new kit and mixed with Q-P images of the base kit.

In the overall pipeline, a user-taken image of the POC test is first processed by a custom instance-segmentation model that automatically corrects orientation and perspective, segments the membrane region from the housing and background, and extracts individual zones (which contain domain-invariant test and control bands) (Fig. 2a). (To assess the accuracy of automated membrane segmentation, we measured the intersection over union scores, i.e., IoU, between the segmented membrane and the manually annotated ground-truth membrane region, with high scores of 0.89 to 0.93, Supplementary Figs. 1 and 2, and Table 1) Images of zone crops enter a feature-extraction network, which is learned to generate robust feature representation to indicate colored rectangular bands (the form factor seen in the vast majority of LFAs^61,62^), such that the positive can be discriminated from negative cases under diverse conditions (including color, intensity, and width of bands). Then, a classifier is learned to determine the presence or absence of a band in each zone. Finally, the output of the binary classifier is compared to a lookup table containing all combinations of possible zone-level classification results to produce a binary classification at the level of the overall test kit, which is displayed as the interpreted result of the LFA (positive or negative) on the user’s smartphone. In this way, the pipeline is agnostic to any kits with lines. Starting with the input of the arbitrary user image, on 1911 images of test kits, this server-hosted pipeline ran with a mean execution time of 3.55 ± 2.28 s (see “Methods” for details).

**Table 1:** Intersection over Union scores for membrane segmentation.

Kit name	IoU score
Flowflex	0.92
DeepBlue	0.89
Jinwofu	0.90
ACON IgG/IgM	0.93
EcoTest housing 2	0.93

The IoU scores for each of the new kit images was obtained by selecting ten images at random from a labeled pool of 30 images for training and evaluating the performance on a fixed evaluation set of ten images.

Unlike methods that require de novo training on a new LFA kit, we developed two methods to achieve adaptation requiring only a small number of images of new kits. First, based on the observation that edges associated with bands tend to remain invariant in diverse LFA images, the feature-extraction network is trained to preserve the edge patterns in an image to ensure the underlying feature representation is robust against variations in LFA images. Thus, the latent representations are trained in a manner that such edges can be reconstructed (decoded) (Fig. 2b). This reconstruction objective supplements the standard classification objective and helps the learning of feature representation for effective adaptation. With the assistance of an automatic edge-detection algorithm, the label can be easily obtained (i.e., not requiring manually-assigned labels by experts on validated specimens), and the edge-preservation character can be learned in a self-supervised manner. During training, we use the output of an automatic edge detection algorithm as the ground truth for decoding. Second, during rapid model adaptation, both the feature-extraction network and classifier are updated with a small number of samples. Here, we regularize the feature-extraction network by performing supervised contrastive learning among the mixture of images from the new LFA kit and the base LFA kit while the classification objective helps update the classifier. In this way, the whole model avoids overfitting to the limited images of the new LFA kit (only 10 to 20) (Fig. 2c).

#### Automated extraction of region of interest from raw user images

The instance segmentation module corrected for skew and extracted the zones from the images of the POC LFA kit. This module first detected the orientation of the kit and carried out perspective correction using the predicted segmentation mask of the LFA kit (Supplementary Fig. 1). This mask was generated by using Mask R-CNN^63^, an instance segmentation model. The kit membrane from the perspective-corrected image was then localized, and individual test zones were cropped out using the kit-specific dimensions listed in a JSON file. For this study, the test-specific dimensions, such as kit height, kit width, membrane width, membrane height, and zone dimensions, were measured from images of LFA kits using Adobe Photoshop v21.0.2 and saved as a JSON file. These dimensions could be directly provided by the kit manufacturers in the future. Further details on image acquisition and processing based on Mask R-CNN are provided in Supplementary Methods.

#### Pre-training of feature extractor with self-supervised learning

The cropped zones of the base LFA kit were used to pre-train a deep neural network feature extractor. The model uses the Mean Squared Error (MSE) between the decoder output (the reconstructed image) and the automatically generated ground truth edge-enhanced image, and the Cross-Entropy (CE) between the classifier output and the ground truth class label as the losses. For the base kit, the number of labeled images was sufficient so that both the classification and the edge-enhanced image reconstruction tasks were carried out to learn a good feature extractor. Thus, as shown in Fig. 2b, output features of each cropped zone are sent to both the classifier and the decoder. For the binary classifier, two specific prototypes are learned and associated with the positive and negative classes using CE loss. In this way, the binary classifier outputs ‘0’ or ‘1’ to denote the absence or presence of the band in the cropped zone, respectively. The decoder is a stack of convolution layers with learnable convolution kernels. The model uses the CE loss for the classification task and the MSE between the reconstruction and the automatically reconstructed edge-filtered image to learn the optimal convolution kernel in the decoder for the self-supervised edge reconstruction task. By using the edge-enhanced features, the feature extractor was able to generalize well on new assay kit images, even if the zones were faint.

To generate the ground-truth of the self-supervision task, the model first converted the RGB image into a grayscale image, and then used an edge filter, e.g., Sobel filter^64^, to highlight the pixels in the edge region (if an edge exists) and obtain edge-enhanced image (Sobel filter is a basic image processing algorithm that generates an image emphasizing edges). The edge-filtered images are then normalized between 0 and 1 and set as labels for the self-supervision task.

With the annotated classification label and the self-generated edge detection label, the equally weighted CE loss and MSE were summed up and used as the objective, allowing the feature extractor, classifier, and decoder to be optimized jointly. In this manner, the extracted features were made discriminative and sensitive to the edge region, and the encoded edge information was used for the classification of cropped zone images, including those with faint bands.

Details on selection of hyperparameters and determination of ambiguity using thresholds are provided in Supplementary Methods.

#### Self-supervision to learn domain-invariant representations (edge detection)

**Fig. 3:**
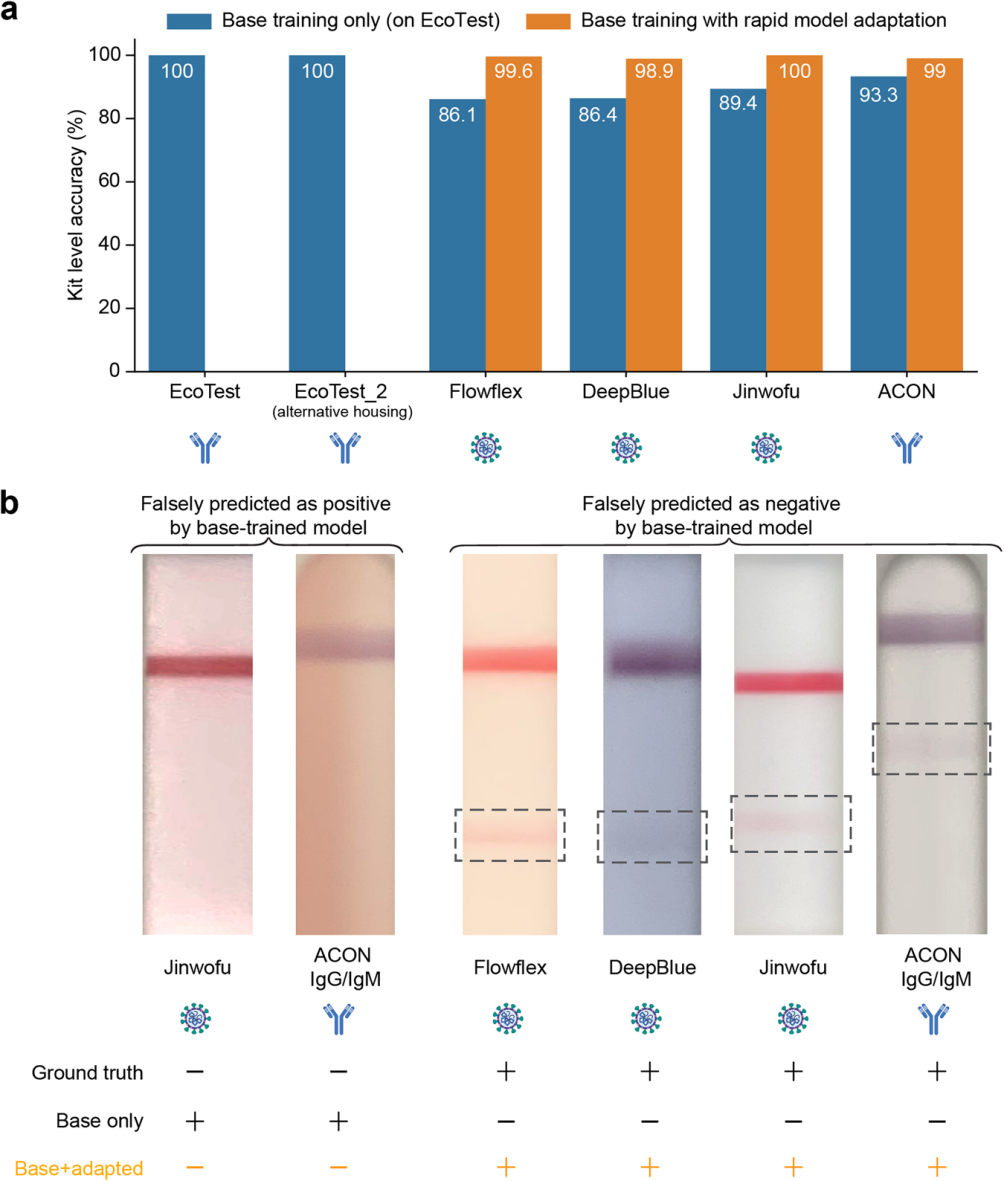
Comparison of kit-level classification accuracy without adaptation (direct testing) and with adaptation. **a** For the direct testing case, the model pre-trained on the base kit was directly applied on each of the new kit’s evaluation dataset. For the adaptation approach, the pre-trained model was adapted to each of the new kits, except for EcoTest housing 2 kit, using 10-shot adaptation (20 zone images) and the performance on their respective evaluation datasets is listed here. (The EcoTest housing 2 kit was identical in all aspects to the base kit expect for the housing, so the direct application of the base model without any adaptation was able to achieve 100% kit-level accuracies.) (*n* = 1 replicate per condition). **b** Images illustrating the challenge for few-shot learning. Sample images of challenging cases that were not classified correctly when using the base model without adaptation and were correctly classified using the adapted models. Shown are both false positives and false negatives (likely due to variations in colors and intensities of membrane background and bands).

The feature extractor is used as a function to obtain the latent representation of cropped test zones. Conventionally, the feature extractor is only learned with CE loss to new LFA kits; however, directly applying such a model trained to new LFA kits will result in low classification accuracy. For example, the model still cannot recognize faint bands (false negatives) but can be confused by the stained membranes and lighting artifacts (false positives) on the new kits (Fig. 3b).

Even though such failure cases can be reduced by training on a large number of relevant examples, acquiring sufficient images on a new LFA kit present a logistical challenge. Hence, in addition to the classification objective, we designed an edge-enhanced image reconstruction task to improve the generalizability of the feature extractor (Fig. 2b). The network was trained to detect the edges of the image pattern (pixels at the junction between the membrane background and the band in the zone) by reconstructing the corresponding edge-enhanced image. Since the edge-enhanced image is normalized, the edges of weak bands can still be highlighted. By using the edge detection algorithm to train the feature extractor in a self-supervised manner, the edge patterns in the image are preserved through the feature extraction process, and the feature-extraction network is trained to capture unique attributes that can be used to recognize faint test kit images and be robust to large domain gaps. In this way, the self-supervision removes the need for complex image calibration pre-processing.

#### Model adaptation to new LFA kits by few-shot domain adaptation

The pre-trained model from a base LFA kit was adapted to a new LFA kit via few-shot adaptation (Fig. 2c). To avoid overfitting to the small number of images of the new kit, we perform pair-wise comparison using supervised contrastive (SupCT) learning^65^ on a mixture of labeled data from the base LFA kit and the new LFA kit. In this way, the positive samples of new kits are trained to be aligned with the positive samples of base kits.

First, we extracted features of the base kit cropped zone images for both positive and negative classes and considered them as anchors. Next, we extracted features from the cropped zone images of the new kit and compared them with all of the anchors using cosine similarity. The feature extractor was then trained to maximize the cosine similarity between features of the same class. For the implementation, we resampled the cropped zone images from the mixed dataset to build episodes and then computed SupCT loss within each episode. Besides minimizing the SupCT loss to refine the feature extractor, a CE loss is used to train the binary classifier on top of the aligned latent features for the new LFA kit. As a comparison to the adaptation strategy, we also performed fine-tuning, which only calculated the CE loss among samples within the episodes for network updating.

### Data acquisition

#### Base model

For base model pre-training, we used expert-labeled images from the AssureTech EcoTest COVID-19 IgG/IgM Antibody Test (base kit), an assay authorized by FDA. For these test kits, serum samples were collected under Mayo Clinic IRB 20-004544 (with informed consent) or shared by the Department of Laboratory Medicine at the University of Washington School of Medicine (Seattle, WA) (informed consent waived due to  the use of discarded samples)^66^. All assay kits were imaged within 10 min of running the test.

Base kit train and validation datasets were gathered using iPhone X at the Mayo Clinic Hospital, Phoenix, AZ. The evaluation dataset images were gathered using three phones by two users: iPhone X, iPhone 7, and Samsung Galaxy J3 (SM-J337V). Care was taken to ensure the kits were imaged under three different ambient lighting conditions (warm white, cool white, and daylight). The training dataset from the base kit consisted of 383 membrane images (674 positive zones and 475 negative zones). An additional 254 membrane images (441 positive zones and 321 negative zones) were used as the validation set for model selection under the fully-supervised classification task. In addition, we used a variational autoencoder^67^ to generate a synthetic dataset composed of 600 zones each of faint positive and negative zones^68^. The synthetic data was mixed with the training dataset for the self-supervised edge-reconstruction task. The performance of the base model is reported on an evaluation set consisting of 102 membrane images (168 positive zones and 138 negative zones) of the base kit.

#### Adapted model

To demonstrate model adaption, we adapted the model to interpret LFAs from five other commercial COVID-19 LFAs. The five LFAs include three antigen tests (ACON Flowflex SARS-CoV-2 Antigen Rapid Test, Anhui DeepBlue SARS-CoV-2 Antigen Test, and Jinwofu SARS-CoV-2 Antigen Rapid Test), one antibody test (ACON SARS-CoV-2 IgG/IgM Antibody Test), and an AssureTech EcoTest COVID-19 IgG/IgM Antibody Test kit that uses a different housing (denoted in the paper as ‘EcoTest (housing 2)’) but retains use of the same LFA membrane. Of the five test kits, the ACON Flowflex antigen test and the AssureTech EcoTest antibody test have been authorized by the FDA. Like almost all commercial LFAs, these kits share rectangular control and test bands, but differ in kit housing dimensions and membrane dimensions, as well as number, spacing, and color of bands (kit-specific dimensions shown in Supplementary Table 3). For these test kits, nasopharyngeal swabs from Mayo Clinic Hospital patients were heat fixed and run for the antigen tests (Mayo Clinic IRB 20-010688). All assay kits were imaged within 10 min of running the test.

New test kit training and evaluation sets were gathered using iPhone X at the Mayo Clinic Hospital, Phoenix, AZ. For the ACON Flowflex SARS-CoV-2 Antigen Rapid Test and the ACON SARS-CoV-2 IgG/IgM Antibody Test specifically, a subset of images taken by untrained users as part of a COVID-19 drive-through study conducted by the Mayo Clinic Hospital (see “COVID-19 drive-through study” for study details) were added to the training dataset. Details regarding the dataset for the five new kits for evaluation are provided in Supplementary Table 4.

### Model evaluation

#### COVID-19 drive-through study

Individuals undergoing standard of care SARS-CoV-2 testing (*n* = 74) were recruited for a drive-through study in the parking lot of the Mayo Clinic in Arizona for additional antigen or antibody self-testing using rapid test kits and a SafeSwab collection device. Participants spanned a range of ages, and education levels (Supplementary Table 5). Two tents were set up, one for check-in, and one for testing. Specimens from study participants were tested by PCR using either the Abbott *m*2000 or the Abbott Alinity m systems.

##### SafeSwab system

Errors in collecting insufficient or excess biological sample and incorrect sample transfer can lead to invalid or incorrect test results^69^. In order to address this, we have developed the SafeSwab system. SafeSwab is a collection device that allows for integrated sample collection and dispensing. After using a standard lancet, the absorbent tip of the SafeSwab can be used to collect a fingerstick blood sample. Or, the swab tip can be extended to reveal ~1 cm of swab surface to collect a nasal sample. Distal to the tip is a reservoir, which can be filled with any buffer to suit the test being performed. Twisting the reservoir releases the buffer to flow down the barrel, carrying the sample out of the absorbent tip. When held over a rapid testing device, the sample can be placed directly into the sample inlet. This device simplifies the sample collection and transfer process and could greatly reduce incidences of operation-related error. The user acceptability of the SafeSwab device was ascertained by asking participants of the drive-through testing study to fill out a user survey after testing themselves using either the ACON Flowflex SARS-CoV-2 Antigen Rapid Test or the ACON SARS-CoV-2 IgG/IgM Antibody Test.

##### Antigen testing population

Participants in the antigen testing arm were recruited via the drive-through COVID-19 testing site at an academic hospital campus. After providing informed consent (IRB protocol 20-010688), participants were asked to remain in their vehicle and were provided with a small tray containing a Flowflex™ SARS-CoV-2 Antigen Rapid Test cartridge (ACON Laboratories), a SafeSwab pre-filled with Flowflex™ SARS-CoV-2 Antigen Rapid Test buffer (ACON), and a mobile phone with the Safe Health Systems HealthCheck application installed.

##### Antibody testing population

Participants in the antibody testing arm were contacted 2 weeks after a positive PCR result and invited to return ≥3 weeks from their positive PCR result and ≥2 weeks from symptom resolution. After informed consent (IRB protocol 20-004544), participants were asked to remain in their vehicle (to simulate a home environment) and were provided with a small tray containing an ACON SARS-CoV-2 IgG/IgM Rapid Test cartridge (ACON), a custom sample collector pre-filled with ACON SARS-CoV-2 IgG/IgM Rapid Test buffer, alcohol prep pad, lancet, bandage, and mobile phone.

##### Self-testing

Participants in both studies used the Safe HealthCheck phone application to complete the testing process. First, the app prompts the user to scan a QR code on the test cartridge pouch, initiating the cognate animated instructional video (motion504, Minneapolis, MN). The video shows the user the entire testing process. After the video, still images from the video instructions with accompanying text take the user through the testing process step by step on subsequent screens. The app includes a built-in timer and subsequent prompt for the user to take a picture of the cartridge (also via the app). The test image is sent to an Amazon Web Server where it is processed by the AutoAdapt POC image analysis algorithm. During the study, the result was not returned to the user but was stored in a de-identified database for documentation purposes.

##### Survey

Each participant was asked to complete a usability study at the end of their experience. During the survey, participants were asked to provide their age and maximum educational level and to answer questions anonymously.

##### Training set

The training set for rapid model adaption consisted of 14 randomly selected test kits (11 Flowflex antigen, 3 ACON antibody tests) imaged by staff during study training.

#### Comparative assessment study

In this comparative assessment study, we compared the interpretation accuracy of the AutoAdapt POC with untrained users and an expert user. 25 untrained users were recruited through word of mouth and via signs posted outside a Mayo Clinic collaborative laboratory at the Arizona State University Health Futures Center. The participants spanned a range of ages and education levels (Supplementary Table 6).

After obtaining consent, participants were led to a room where they perused the ACON FlowFlex Antigen test kit IFU (Instructions for Use) (Supplementary Fig. 3). They were then provided a set of tests that  had been run with known titers (categorized in this study as high, medium, and low, based on kit reported limit of detection and corresponding to virus titers of 25,000, 17,500, and <7500 TCID_50_/mL, respectively (Supplementary Table 7)) that correspond to a range of band intensities on the ACON FlowFlex test. Participants were asked, referring to the IFU, to interpret the bands as a test result. All participants were asked to observe the IFU for reference. Participants then verbally declared the results, which were recorded by study staff. Participants then took an image of the kit using a study phone. The image was sent to the cloud server for interpretation using AutoAdapt POC (results from AutoAdapt POC were stored only in the server and were not available to both the participants or the expert). Each participant interpreted up to eight tests (details in Supplementary Table 8). In the final analysis, two users were determined to not pass the English-speaking criteria.

Independently, a trained member of the study staff (a registered nurse) also interpreted each rapid test. When making the interpretation, the study staff was not aware of the titers on each rapid test or interpretations by the machine-learning algorithm.

#### HIV rapid test kit images

In a previously published study^8^, 60 fieldworkers in rural South Africa took 4443 images of a HIV rapid test kit (ABON HIV 1/2/O Tri-Line Human Immunodeficiency Virus Rapid Test Device). This dataset was utilized to validate our approach on an application besides COVID-19. Using this dataset, we rapidly adapted both the pre-trained instance segmentation model and the classifier to the ABON test kits using 75 images and 40 images (i.e., 20-shot), respectively.

### Reporting summary

Further information on research design is available in the Nature Portfolio Reporting Summary linked to this article.

## Results

### Accuracy of prediction for five new COVID-19 tests

We assessed the performance of this few-shot adaptation strategy on five COVID-19 LFA kits that exhibited different numbers of test lines and form factors. The performance using only the base model (i.e., the pre-trained feature extractor) was assessed on the new kits, compared to the rapid adaptation method using 10 shots (20 zone images). On a similar LFA kit where the membrane region stayed the same but only the housing was changed, our pipeline with flexible image pre-processing (i.e., automatically extracting zones) and a pre-trained base model supplemented with edge reconstruction produced perfect accuracy (i.e., the EcoTest alternative housing, Fig. 3a). On four additional COVID-19 diverse test kits, the few-shot adaptation strategy consistently resulted in a substantial improvement in performance (i.e., 99 to 100% accuracy in interpreting the overall test kit, compared to 86 to 93%) by including only a few training images of the new LFA kits (Fig. 3a), with no loss in accuracy.

Moreover, we looked at images from new LFA kits that were classified incorrectly when directly applying the base model. These images included kits interpreted incorrectly as positive and incorrectly as negative, for both COVID-19 antigen and antibody tests. Examples included faint lines, shadows, missing control lines, and blurred images (Fig. 3b). With only a few training images of a new LFA kit, the rapidly adapted model correctly interpreted these new LFA kits compared to ground truth (Fig. 3b).

**Fig. 4:**
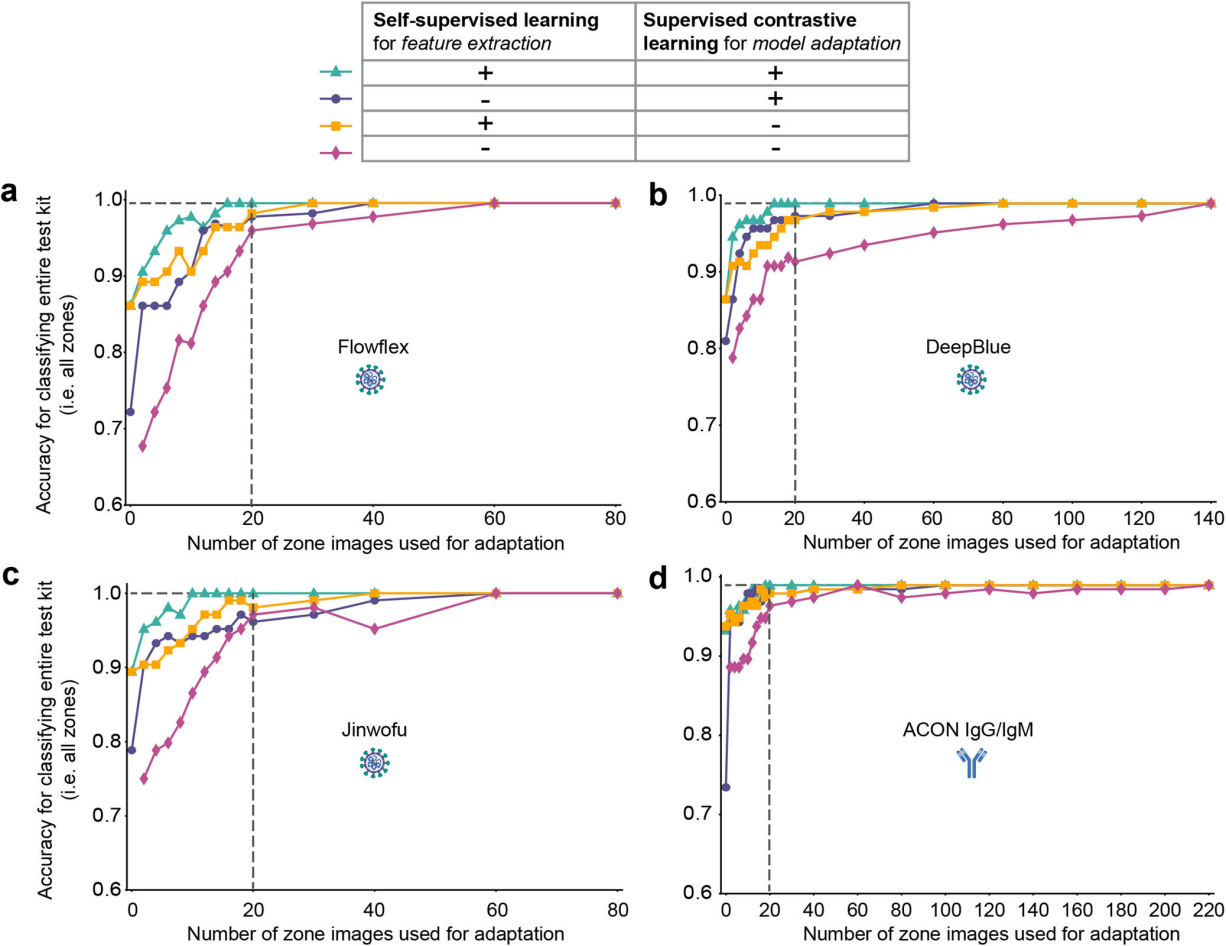
Classification accuracies for four new COVID-19 LFA kits with different numbers of training images used and with ablated models. Ablation studies were carried out to analyze the relative contributions of self-supervised learning for feature extraction and supervised contrastive learning for adaptation. Each model was evaluated by varying the number of images used in the adaptation. Accuracy scores reported for four new assay kits, Flowflex (**a**), DeepBlue (**b**), Jinwofu (**c**), and ACON IgG/IgM (**d**). (The EcoTest housing 2 kit was identical in all aspects to the base kit expect for the housing, so the direct application of the base model without any adaptation was able to achieve 100% kit-level accuracies). The maximum accuracy indicates the upper bound attained by training a model from scratch using all training images for each kit.

We examined further how many training images of a new LFA kit were required in order to achieve high accuracy. Moreover, we performed this assessment with an ablation study that eliminated one or both of two components in the pipeline: self-supervised pre-training of feature extractor (i.e., edge reconstruction) and supervised contrastive learning during rapid model adaptation. Meanwhile, the approach with self-supervised pre-training but without supervised contrastive learning can be considered a fine-tuning process that uses a standard classification objective. For all approaches, images from the base and new kits were combined for training, with the same data sampling strategy used to ensure a fair comparison. For each new kit, a random set of images were selected from the training dataset for model adaptation, and the performance of the trained model was validated against a separate evaluation dataset; we compared the accuracy against that achieved by using the entire dataset of available training images, which was the upper bound of performance. (We didn’t select weak images as we wanted the training process to be truly random. Sampling different sets of images yielded consistent results, as evidenced in Supplementary Table 9). The results showed that for each of four new COVID-19 kits (Flowflex, DeepBlue, Jinwofu, and ACON IgG/IgM), the model achieved maximum classification accuracy using just 16, 14, 10, and 18 zone images, respectively (Fig. 4). For example, for Flowflex, the model required only eight zone images per class (16 zone images) to reach the same performance (99.6%) as a model trained from scratch using all available training data (200 zone images).

**Fig. 5:**
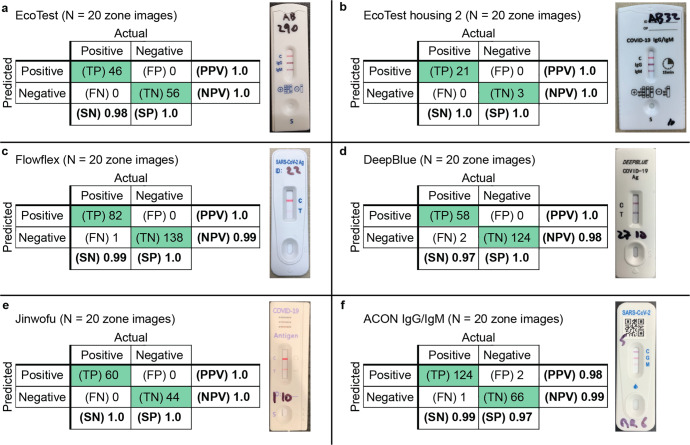
Confusion matrices for the pipeline applied on the evaluation dataset. The model used both self-supervised pre-training of feature extractor (incorporating edge detection) and supervised contrastive adaptation. Confusion matrices are shown for (**a**) base kit, and new kits (**b**–**f**).

Importantly, the results illustrated the importance of both self-supervised pre-training component and supervised contrastive (SupCT) loss component, with the combination of both components producing high accuracy requiring the smallest number of training images of a new test kit (Fig. 4). First, under our procedure for self-supervised pre-training of the feature extractor, the extracted features are sensitive to domain-invariant edges even as they are presented in the new rapid tests. For example, the ACON IgG/IgM kit exhibited the highest frequency of faint bands of all the new COVID-19 LFAs, but our approach by training with only nine images of each class (18 zone images) exhibited the same accuracy as by using the entire training dataset (Fig. 4). (Without self-supervised pre-training, 140 images, with SupCT loss, and 200 images, without SupCT loss, of each class were required comparable performance.) Second, supervised contrastive learning greatly reduced the number of images required. For example, adaptation without this component required at least 80 images for similar performance. Finally, direct testing of the model pre-trained on the base kit (i.e., zero-shot adaptation) was higher when trained using self-supervision than when trained using only the classification objective. The confusion matrices that summarize the overall performance for interpreting the new test kits using *n* = 20 images of new test kits are shown in Fig. 5. (Also, the results were improved by calculating a probability score for predicting the ground truth and classifying images with scores below a threshold to be ambiguous rather than positive or negative, see “Methods” for details.)

### Validation on images taken by users in a COVID-19 drive-through study

**Fig. 6:**
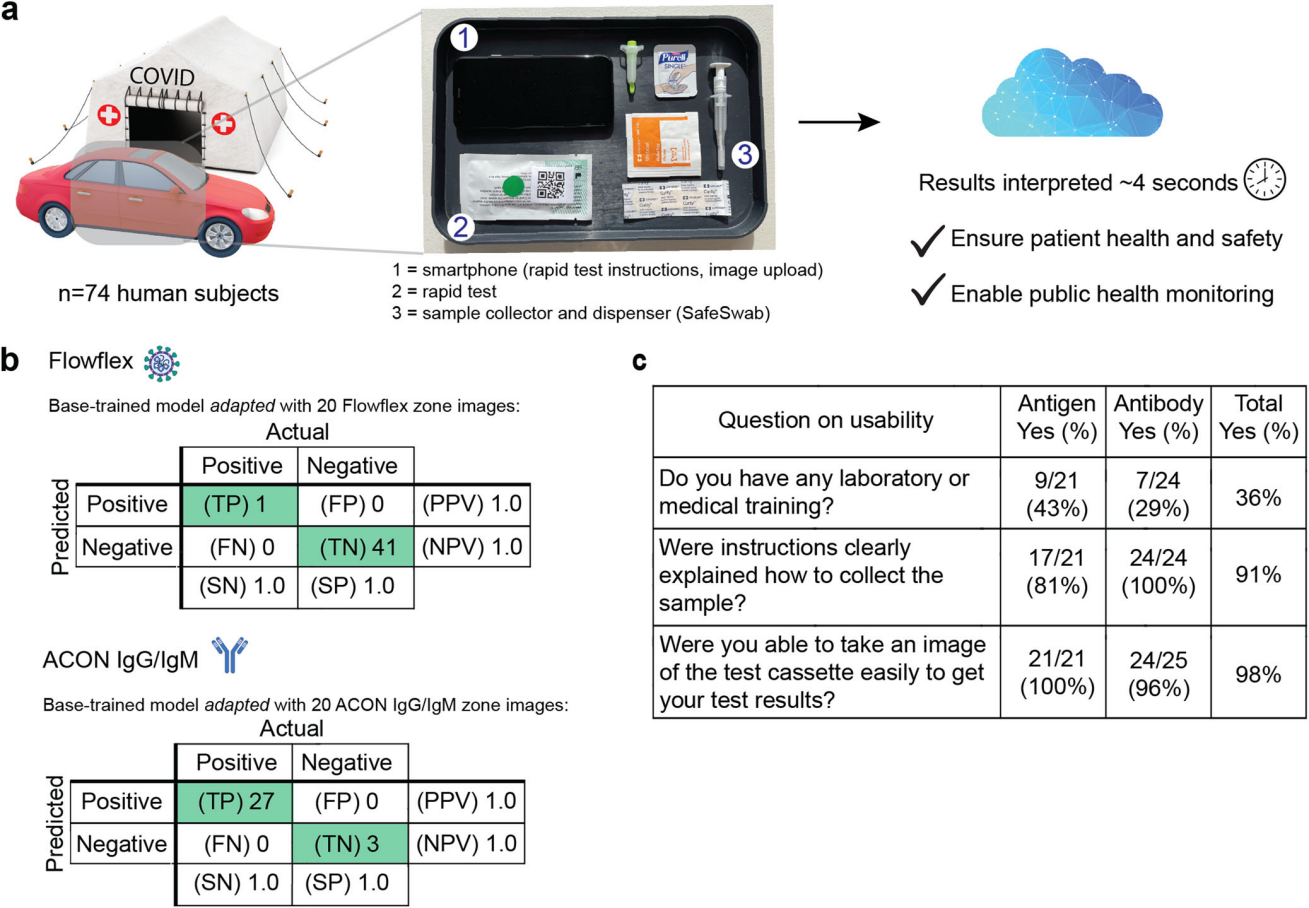
Application of algorithm for interpreting rapid SARS-CoV-2 antigen and antibody tests from 74 untrained users in a COVID-19 drive-through field study. **a** Setup of COVID-19 drive-through field tent, with users performing the tests and collecting the images in their cars. **b** Confusion matrices showing performance of the rapidly adapted algorithm on both rapid antigen and antibody COVID-19 tests. **c** Results of surveying 45 untrained users on usability of overall process. Graphics in (**a**) are from an open-source repository.

We evaluated the AutoAdapt POC algorithm on images of rapid antigen and antibody tests taken by 74 untrained users during a COVID-19 drive-through study (Fig. 6a). In their cars, participants performed and took an image of either the Flowflex™ SARS-CoV-2 Antigen Rapid Test or the ACON® COVID-19 IgG/IgM Rapid Test using a smartphone. In total, 44 images were collected of the Flowflex Antigen test, and 30 images of the ACON antibody test. Two test kits produced invalid results (i.e., no control band present). The rapidly adapted model identified with 100% accuracy the results for all 42 Flowflex antigen tests and all 30 ACON antibody tests in the evaluation set (Fig. 6b). Moreover, in a usability survey of the untrained users, while only 36% reported as having prior laboratory or medical training, 98% of participants (45 out of 46) reported they were able to successfully take an image of the test kit (Fig. 6c).

### Comparison of visual interpretation with AutoAdapt POC

Next, we performed a comparative assessment of the AutoAdapt POC algorithm with traditional visual interpretation by experts and non-experts. 23 non-experts interpreted ACON Flowflex test kits pre-run using contrived samples (87 contrived positive and 18 contrived negative).

Over the 105 test kits (Table 2), the AutoAdapt POC algorithm correctly interpreted all 105 kits (100% accuracy), which matched visual interpretation by an expert. Compared to non-experts, the algorithm had one false negative reading with a low positive test visually interpreted by the participant as negative (99% accuracy) (Supplementary Fig. 4).

**Table 2:** Comparison of visually interpreted results and results predicted by the AutoAdapt POC algorithm.

Specimen used on test kit	Number of tests	Visual interpretation by non-expert (pos/neg)	Visual interpretation by expert (pos/neg)	Automated interpretation by AutoAdapt POC algorithm (pos/neg)	Concordant interpretation (Visual non-expert vs. AutoAdapt POC)	Discordant interpretation (Visual non-expert vs. AutoAdapt POC)
Contrived positive	87	86/1	87/0	87/0	86	1
High positive titer	31	31/0	31/0	31/0	31	0
Medium positive titer	27	27/0	27/0	27/0	27	0
Low positive titer	29	28/1	29/0	29/0	28	1
Contrived negative	18	0/18	0/18	0/18	18	0
Total	105	86/19	87/18	87/18	104	1

Contrived samples were provided by ACON. Titers of high, medium, and low, correspond to virus titers of 25,000, 17,500, and <7500 TCID_50_/mL, respectively. Tests with both visual interpretations and digital reads were included in the analysis.

### Validation of approach on HIV rapid test kit images

**Fig. 7:**
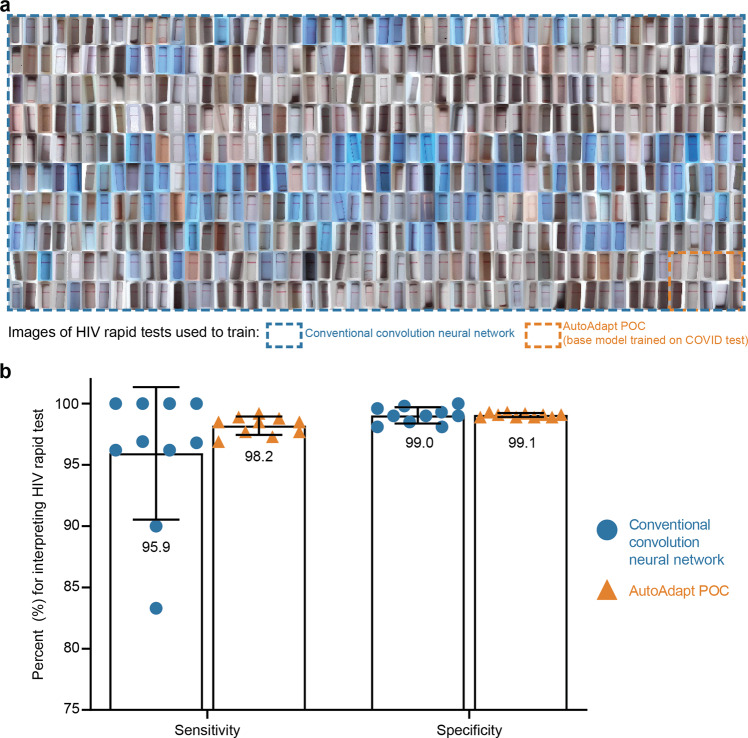
Comparison of AutoAdapt POC base-trained with a COVID-19 test and rapidly adapted to HIV rapid tests, with a traditional convolutional neural network trained to the HIV rapid tests. **a** Training dataset size comparison. The 10 images within the red border demonstrate the number of training images needed by the AutoAdapt POC approach which is a fraction (1/50th) of the images needed by the conventional approach (500 images shown enclosed with the black border). **b** Bar plot comparing the mean sensitivity and specificity scores across conventional training and AutoAdapt POC (error bars represent standard deviation). Data for conventional convolution neural network is from Fig. 3b in a manuscript published in *Nature Medicine*^8^ (*n* = 10 replicates per condition).

Finally, we tested the robustness of the AutoAdapt POC algorithm on a LFA test kit designed for an application other than COVID-19 (Fig. 7). For model adaption and evaluation, we used images of a HIV rapid test kit (ABON HIV 1/2/O Tri-Line Human Immunodeficiency Virus Rapid Test Device) captured in a previously published study^8^.

First, for automated membrane extraction, the adapted instance segmentation model achieved a mean IoU score of 0.927 on 10 random test images of HIV test kits. Next, we carried out 10-fold cross validation (as previously performed^8^), using 40 randomly selected images (20 positive and 20 negative) for rapid adaptation and all remaining images for the test set (262 positive and 4140 negative). With 75 training images and 4402 evaluation images, the performances of each of the 10 folds of the adapter classifier in the AutoAdapt POC algorithm were successively tabulated (Table 3), with mean sensitivity and specificity of 98.2% ± 0.8% and 99.1% ± 0.2%. These values exceeded the mean values of 95.9% ± 5.1% and 99.0 ± 0.6%, respectively, in a previously tested traditional convolution network^8^ that required 3998 training images and assessed on 445 evaluation images. The AutoAdapt POC algorithm also exhibited lower variance. This result highlights the utility of the few-shot domain adaptation for achieving classification performance exceeding a traditional convolution neural network trained from scratch on ~4000 images, by using just 75 images for adaptation (Supplementary Table 10 shows the confusion matrix of the best performing model, i.e., fold 6).

**Table 3:** Performance of AutoAdapt POC approach across 10 folds on ABON HIV image dataset.

Fold	Sensitivity (%)	Specificity (%)
1	98.79	99.25
2	98.38	99.22
3	97.98	99.05
4	97.14	99.05
5	97.98	99.05
6	98.85	99.25
7	96.73	98.96
8	98.79	98.93
9	99.19	98.88
10	97.98	98.86
Average	98.18 ± 0.79	99.05 ± 0.15

Table listing the sensitivity and specificity values of 10 different folds on the evaluation dataset of 4402 images.

## Discussion

We have described the development of AutoAdapt POC, an approach for rapidly adapting machine-learning models for interpreting POC LFAs that span diverse analytical targets, number of test and control bands, and housing form factors. This adaptation can be carried out using a much smaller subset of images than required for training the base model. Compared to de novo training on every new assay kit, this reduction in the number of images was achieved by adopting a modular approach to the machine-learning pipeline: starting from an image of the kit, the perspective-corrected membrane and individual zones were extracted, followed by the extraction of the features preserving edge information, and finally a binary output which indicated whether a band was present in the cropped zone. A robust feature extractor was critical for handling challenging images in LFA kits, like those with faint or partially formed lines, with a self-supervised learning approach to reconstruct edge-enhanced images. The algorithm was also shown to match the accuracy of results interpreted visually by expert users and was able to correctly interpret a kit that was misinterpreted by a non-expert user, highlighting the utility of such an algorithm as testing becomes more decentralized.

There are limitations to our current approach. Our current workflow requires a user to take an image of an entire test kit to properly orient it for analysis, which reduces the resolution of the images used for classification of test bands. The current algorithm does not specifically handle blood staining of antibody rapid tests. Future work will focus on addressing these limitations as well as validation on a wider variety of rapid tests, bands of multiple colors as in some home urinalysis kits, and generalization to rapid kits beyond rectangular bands (e.g., vertical flow assays). We will also explore quantifying the intensity of the test band (based on previous studies^70–72^) to expand applications beyond binary detection.

More broadly, the results here point to the potential of applying few-shot learning (which is increasingly prevalent in a host of non-medical imaging applications) to classifying medical images in which requisition of validated training images poses considerable clinical challenges when used in practice and at scale. For diagnostics, this reduction in new training images to achieve assured user interpretation of rapid test images is substantial given the ubiquity of home use of rapid diagnostic tests. Most immediately, the COVID-19 pandemic has thrusted front and forward the need for rapid testing and population surveillance to track and control the spread of the disease in a scalable and timely manner. If effectively implemented, point-of-care testing can vitally contribute to a rapid and effective public health response—as well as patients’ individual safety, privacy, physical health, and mental well-being—by enabling widespread timely testing in a manner that does not overwhelm the limited capacity of testing facilities or provoke social crowding at selected testing sites.

By expediting the process of training a model to newly available rapid diagnostic tests, the AutoAdapt POC approach could facilitate reliable decentralized testing and real-time monitoring of disease prevalence. (We have also built a mockup of a sample dashboard, using R shiny and leaflet libraries, to demonstrate the potential real-time visualization of data generated by the algorithm of this study as collected from smartphones; see Supplementary Fig. 5 and Supplementary Video 1). Over time, scalable approaches to achieve assured user operation and results interpretation as well as reliable data monitoring will be increasingly vital, as patients and consumers will monitor their health via increased self- and home-testing for both infectious diseases and chronic conditions in the oncoming age of digital and precision health.

## Supplementary information

Supplementary Information

Supplementary Movie 1

Supplementary Data 1

Supplementary Data 2

Reporting Summary

Description of Additional Supplementary Files

## Data Availability

All test kits images used for base model training, adaptation, and for the drive-through study are available to download from the following link upon request. https://drive.google.com/drive/folders/1PJRmiCviniQShOJcBxmDFpOCkIscRGZ9?usp=sharing. The dataset used for evaluating the algorithm on an HIV rapid test kits is available here: https://data.ahri.org/index.php/catalog/923^8^. Source data for Fig. 4 is available in Supplementary Data 1. Source data for Fig. 7c is available in Supplementary Data 2. All other data is available from the corresponding author on reasonable request (pending protection of patient privacy).

## Code availability

Custom code used in this study is available on this GitHub repo: https://github.com/smartestapp/smart-ml/. The DOI is provided by Zenodo^73^.
