# Supplementary material for: Rapidly adaptable automated interpretation of point-of-care COVID-19 diagnostics
Source: Commun Med (Lond). 2023 Jun 23;3:91. doi: 10.1038/s43856-023-00312-x (PMC10290128; doi:10.1038/s43856-023-00312-x)
Supplement: Supplementary file 1 — Supplementary Information [file 43856_2023_312_MOESM1_ESM.pdf]

Supplementary Information for:

Rapidly Adaptable Automated Interpretation of Point-of-Care COVID-19 Diagnostics

Siddarth Arumugam[1†]; Jiawei Ma[2†]; Uzay Macar[2]; Guangxing Han[3]; Kathrine McAulay[4]; Darrell Ingram[5]; Alex Ying[1]; Harshit Harpaldas[1], Terry Chern[1], Kenta Reilly[4], David A. M. Colburn[1]; Robert Stanciu[1]; Craig Duffy[5], Ashley Williams[5], Thomas Grys[4]; Shih-Fu Chang[2,3*‡]; Samuel K. Sia[1*#]

[1] Department of Biomedical Engineering, Columbia University, New York, NY 10027, USA

[2] Department of Computer Science, Columbia University, New York, NY 10027, USA

[3] Department of Electrical Engineering, Columbia University, New York, NY 10027, USA

[4] Department of Laboratory Medicine and Pathology, Mayo Clinic, Phoenix, AZ 85054, USA

[5] Safe Health Systems, Inc., Los Angeles, CA 90036, USA

[†] These authors contributed equally.

* These authors jointly supervised this work.

[‡] sc250@columbia.edu

[#] ss2735@columbia.edu

A. **Supplementary Tables**

B. **Supplementary Figures**

C. **Supplementary Methods**

D. **Supplementary References**

A. Supplementary Tables

Supplementary Table 1. Comparison of ML approaches for analyzing diagnostic test image data.

**Assay format that generates diagnostic test image data**	**Hardware complexity**	**Exemplary reference**	**ML model**	**Number of training images**	**Real-time operation requirement**	**Performance**
Microfluidic	High	[1]	CNN	15,057		
Microfluidic	High	[2]	Domain adaptation, episodic training (no few-shot learning)	1000s of unlabeled images		
LFA	Low	[3]	CNN	4000 to 6000	Manual user alignment of images	95% sensitivity, 95% specificity
LFA	Low	[4]	CNN	498		100% sensitivity, 96% specificity
LFA	Low	[5]	CNN	~3000		98.9% sensitivity
LFA	***Low***	***Current study***	***Few-shot learning, with episodic training***	***20***	***Automated image pre-processing*** (demonstrated with data collection from field site)	***99% sensitivity, 99% specificity***

Supplementary Table 2. Smartphone-based diagnostics comparison table

Test type	Hardware requirement category	Specific hardware requirement	No. of training images from target domain	Real-time result generation (within seconds)	References
Paper-based assays	External kit/phone holder and custom optics	3D printed enclosure with embedded lighting system	2291	Yes	[6]
		3D printed enclosure with LEDs and external lens	~200	Yes	[1,7]
		Enclosure with real-time camera reader	N/A	Yes	[8]
	External kit/phone holder	3D printed platform to hold smartphone and rapid test kit	N/A	Yes	[9]
		Black cardboard box to hold test kits and smartphone	N/A	No	[10]
	LFA casette modifications	Cassette sticker for detection	N/A	Yes	[11]
	No hardware requirement	None	650	Yes	[12,13]
		None	N/A	Yes	[14]
		None	476	Yes	[15]
		None	498	Yes	[4]
		None	~3000	Yes	[5]
		None	4000 to 6000	Yes	[3]
		None	***10 to 20***	***Yes***	***This work***

Supplementary Table 3. Assay kit parameters. Summary of manually measured parameters for each of the assay kits.

Kit name	Designation	No. of zones per kit	Kit aspect ratio	Membrane aspect ratio	Zone aspect ratio
EcoTest	Base	3	0.29	0.21	3.23
Flowflex	New	2	0.28	0.22	2.94
DeepBlue	New	2	0.28	0.19	2.17
Jinwofu	New	2	0.30	0.21	3.03
ACON IgG/IgM	New	3	0.28	0.21	2.86
EcoTest housing 2	New	3	0.53	0.21	3.13

Supplementary Table 4. Dataset split of training and evaluation sets for 5 new kits. The train and evaluation dataset sizes in terms of cropped zone images and membranes for the five new assay kits.

	Flowflex		DeepBlue		Jinwofu		ACON IgG/IgM		EcoTest housing 2	
	Zone	Memb.	Zone	Memb.	Zone	Memb.	Zone	Memb.	Zone	Memb.
Positive class (train)	149	50	125	25	146	50	182	63	20	5
Negative class (train)	51	49	75	75	54	46	118	37	10	5
Positive class (evaluation)	304	83	244	60	164	60	386	126	66	21
Negative class (evaluation)	142	138	124	124	44	44	193	67	6	3

Supplementary Table 5. Patient survey results after COVID-19 drive through Study. Note that 21 (53%) antigen testing participants and 25 (78%) antibody participants opted to complete the survey.

	**Antigen**	**Antibody**	**Total**
**Education Level**	**Number (%)**	**Number (%)**	**Number (%)**
Doctorate	1 (5%)	0 (0%)	1 (2%)
MS	1 (5%)	5 (20%)	6 (13%)
BS/BN	16 (76%)	14 (56%)	30 (65%)
High School or below	3 (14%)	6 (24%)	9 (22%)
**Age**	**Number (%)**	**Number (%)**	**Number (%)**
18-29	4 (19%)	0 (0%)	4 (9%)
30-39	4 (19%)	4 (16%)	8 (17%)
40-49	1 (5%)	4 (16%)	5 (11%)
50-59	6 (29%)	5 (20%)	11 (24%)
60-69	3 (14%)	9 (36%)	12 (26%)
70-79	3 (14%)	3 (12%)	6 (13%)

Supplementary Table 6. Patient demographics for comparative assessment (n=23).

**Education Level**	**Number (%)**
Doctorate	5 (22%)
MS	7 (30%)
BS/BN	6 (26%)
High School or below	4 (17%)
Declined to answer	1 (4%)
**Age**	**Number (%)**
18-29	8 (35%)
30-39	5 (22%)
40-49	4 (17%)
50-59	5 (22%)
60-69	1 (4%)
70-79	0 (0%)

Supplementary Table 7. Details on contrived specimens utilized in comparative assessment study. Dilutions are based off the limit of detection (LoD) for the ACON FlowFlex test as reported the manufacturer (2500 $TCID_{50}$/mL).

**Specimen**	**Dilution Level**	**Concentration ($TCID_{50}$/mL)**
High positive titer	10 x LoD	25000
Medium positive titer	7 x LoD	17500
Low positive titer	3 x LoD	7500
	2 x LoD	5000
	1x LoD	2500

Supplementary Table 8. Number of kits visually interpreted by participants in comparative analysis. Two participants were excluded due to failure to meet the English speaking requirement.

Participant number	Number of test kits compared
1	4
2	4
3	4
4	4
5	4
6	3
7	3
8	2
9	2
10	1
11	6
12	6
13	5
14	6
17	6
18	6
19	6
20	6
21	4
22	3
23	4
24	4
25	8

Supplementary Table 9. Mean classification accuracy scores using bootstrapping. The mean accuracy values were determined by carrying out 20 trials of bootstrap sampling with 10-shot adaptation (20 zone images) on the new kit's training dataset and evaluating the performance on the evaluation dataset. Data is represented as mean ± standard deviation.

Kit name	Zone accuracy (%)	Kit accuracy (%)
Flowflex	99.6 ± 0.2	99.3 ± 0.3
DeepBlue	99.3 ± 0.2	98.6 ± 0.4
Jinwofu	99.6 ± 0.4	99.2 ± 0.7
ACON IgG/IgM	98.4 ± 0.5	95.3 ± 1.3

Supplementary Table 10. Performance summary of AutoAdapt POC approach on ABON HIV image dataset. Confusion matrix of fold number 6. 9 negative membranes were classified as invalid and are not listed in the table.

Label / Prediction	Positive	Negative
**Positive**	(TP) 259	(FP) 31
**Negative**	(FN) 3	(TN) 4100

Supplementary Table 11. Mean IoU scores using bootstrapping. The mean IoU scores for each of the new kit images, except for the EcoTest housing 2, was obtained by carrying out bootstrap sampling and obtaining three resamples. Each sample had ten randomly chosen images from a labelled pool of 30 images for training and the performance was evaluated on a fixed evaluation set of ten images. Due to a limitation on the available images only a single sampling was used for the EcoTest housing 2. Data is represented as mean ± standard deviation.

**Kit name**	**IoU score**
Flowflex	0.92 ± 0.01
DeepBlue	0.91 ± 0.01
Jinwofu	0.91 ± 0.08
ACON IgG/IgM	0.92 ± 0.01

B. Supplementary Figures

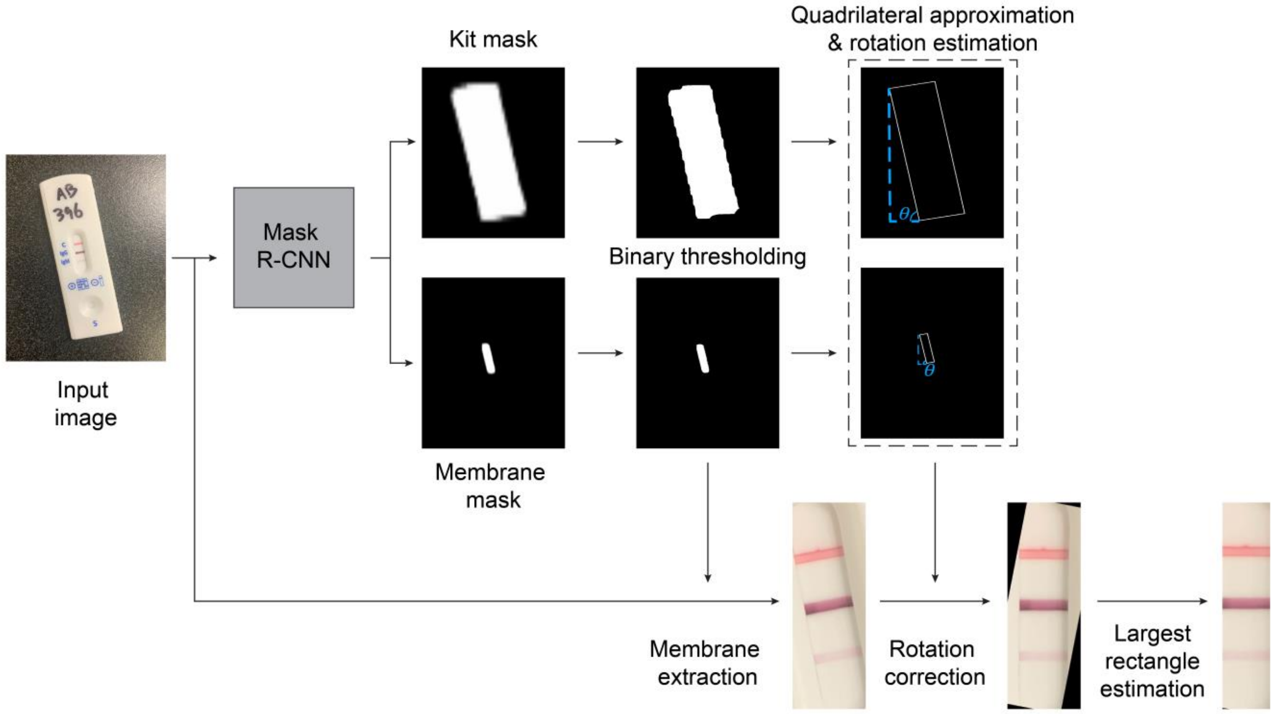

Supplementary Figure 1. Workflow for membrane extraction and rotation correction. The assay kit image is passed through the instance segmentation model Mask R-CNN. The kit and membrane segmentation masks are obtained and binarized. The kit or the membrane mask is used for quadrilateral approximation and rotation estimation in the image, and the membrane mask is used to extract the membrane from the input image. The estimated rotation value is used to correct the perspective of the extracted membrane. Largest rectangle estimation is carried out to remove the black pixels in the rotation-corrected membrane to retain only the relevant pixels

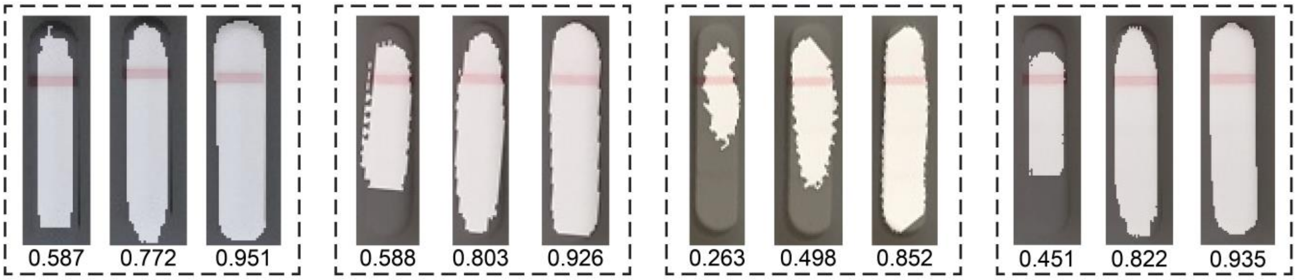

Supplementary Figure 2. Illustration of different IoU scores for membrane segmentation. Images shown are of the EcoTest (base kit) with the membrane segmentation mask overlaid on the zoomed-in image of the membrane. For each membrane image, segmentation masks corresponding to different IoU scores are shown with the score listed below the image.

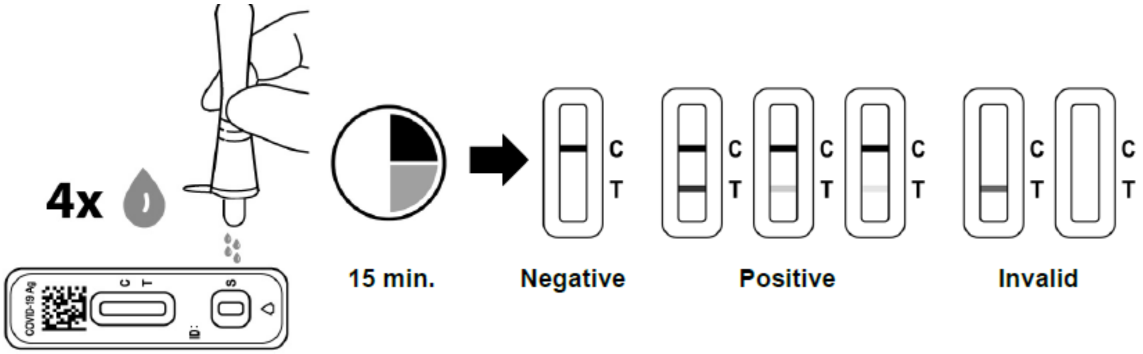

INTERPRETATION OF RESULTS

(Please refer to the illustration above)

NEGATIVE: Only the control line (C) and no test line (T) appears. This means that no SARS-CoV-2 antigen was detected. A negative test result indicates that antigens from the virus that causes COVID-19 were not detected from the specimen. Negative results do not rule out COVID-19. Individuals without symptoms that test negative should be tested again with at least 24 hours and no more than 48 hours between tests. All negative results are considered presumptive, and confirmation with a molecular assay, if necessary for patient management, may be performed.

POSITIVE:* Two red or pink lines appear in the test window, one on the test line position (T) and the other on the control line position (C). A positive test is interpreted as protein antigen from the virus that causes COVID-19 was detected in the specimen. The individual is positive for COVID-19. Test results should be considered in association with the patient's history and epidemiological data (such as local prevalence rates and current outbreak/epicenter locations).

* **NOTE:** The test line (red or pink line) may vary in shade and intensity (light or dark, weak or strong) depending on the concentration of antigen present in the sample. The intensity of the Control line should not be compared to that of the Test line for the interpretation of the test result. Any faint visible red or pink Test line should be interpreted as positive, when the control line (C) is also present.

INVALID: Control line fails to appear. If a line does not appear on the control line position (C) in 30 minutes, the test result is invalid. Re-test with a new Flowflex COVID-19 Antigen Home Test.

Supplementary Figure 3. Excerpt for Instructions for Use for ACON Flowflex. Non-experts consulted these instructions before visual interpretation of the bands, in the comparative assessment study. (https://www.fda.gov/media/152698/download)

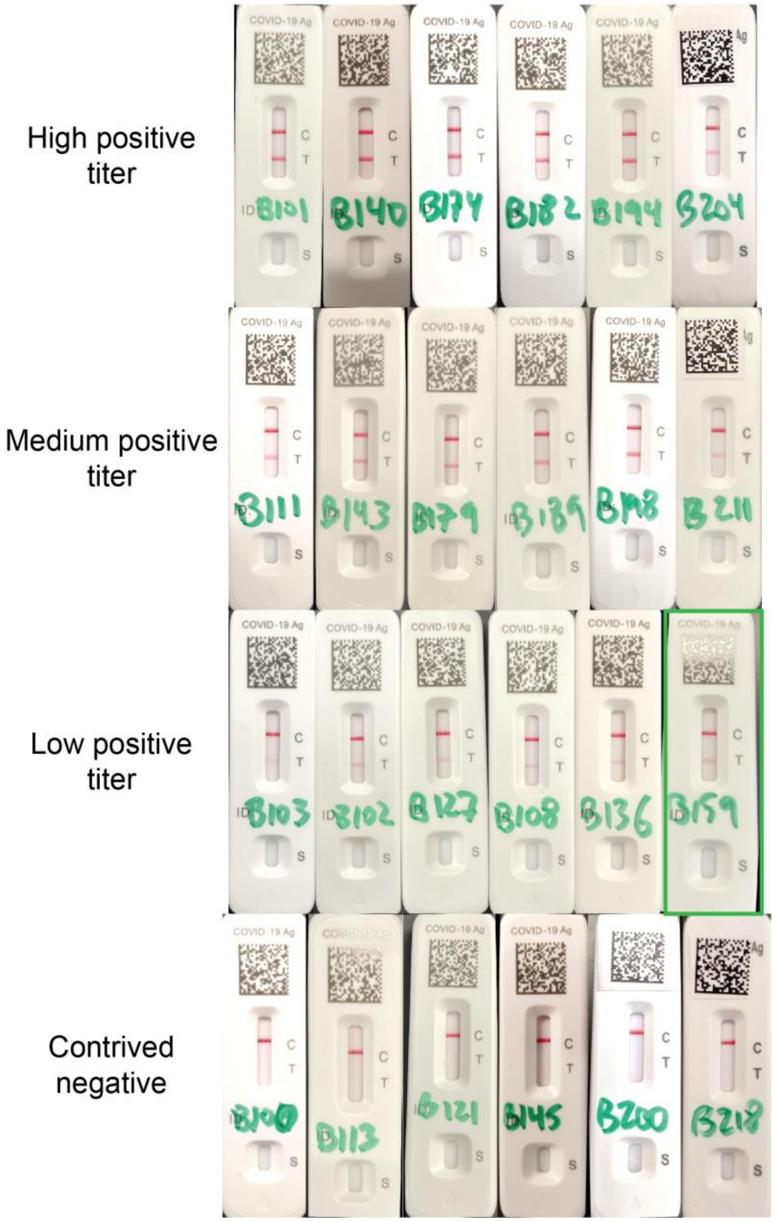

Supplementary Figure 4: Subset of test kit images captured in comparative analysis study using contrived samples. Titers of “high”, “medium”, and “low, corresponded to target tissue culture infectious dose of 25,000, 17,500, and <7500 dilution factors, respectively. Highlighted in green is the test kit that triggered the false-negative reading with the algorithm compared to non-expert (interpreted as negative by the non-expert)

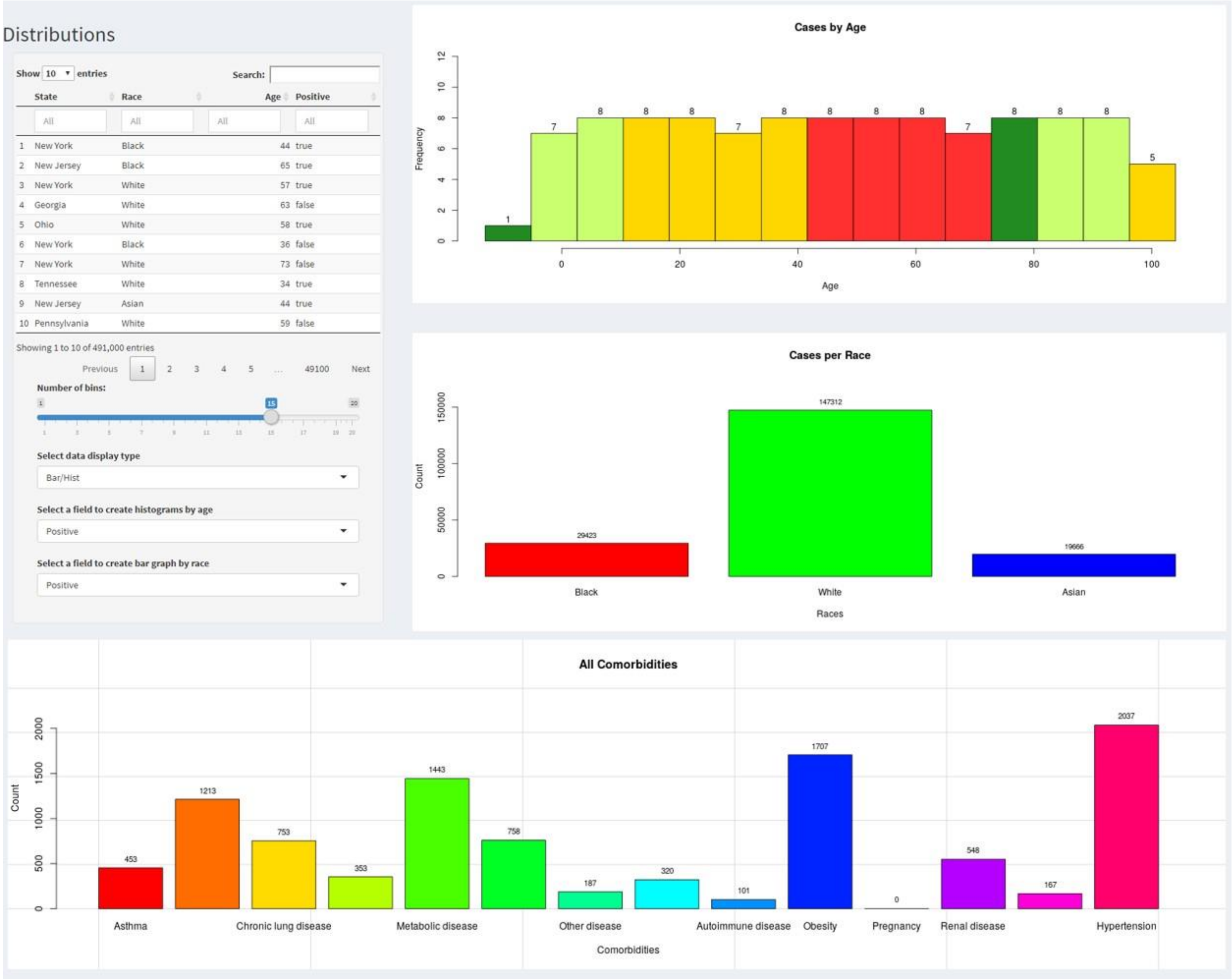

Supplementary Figure 5. Sample data display dashboard for real-time population disease surveillance. The sample dashboard displays graphical plots for tracking user demographic data in real-time using the results generated by the AutoAdapt POC pipeline.

C. Supplementary Methods

Image acquisition and processing based on Mask R-CNN

The image processing workflow starts with an image of the assay kit being taken by the user through the SMARTtest application[13] in a fixed portrait orientation. This image is saved in an AWS S3 bucket as an JPEG image from the frontend, and the corresponding URL is sent to the AWS Lambda Function. The function reads the image data, stores the original resolution image in a copy, and resizes the image while preserving the aspect ratio by capping the height of the image to a maximum of 800 pixels. The membrane is localized in the resized image using the instance segmentation model Mask RCNN (**Supplementary Figure 1**), and the predicted bounding box coordinates in the resized image are then transformed to the corresponding coordinates in the image of the original resolution to get the highest possible resolution of the membrane which is then sent to the classifier.

Mask R-CNN[16] builds on top of the preceding Faster R-CNN[17] and Fast R-CNN[18] models and combines them with a fully-convolutional network (FCN) and introduces object mask prediction (i.e., segmentation[19]) in parallel to bounding box regression. Given an input image, the model extracts feature maps via a pretrained deep neural network (e.g., VGG16), and subsequently passes these in parallel through a ROI-specialized pooling layer followed by several fully-connected layers and an FCN. The instance segmentation model has been trained for two object classes: the kit and the membrane. The model outputs i) detection scores, ii) bounding boxes, and iii) segmentation masks of a maximum of 100 objects. The bounding box

defines a rectangular area that contains the assay kit or the membrane. The segmentation mask includes all the pixels that correspond to the actual area of the assay kit or the membrane and do not necessarily have to be rectangular in shape. From all the detected objects we retain information for a kit and membrane object with the highest detection score greater than 0.9. **Supplementary Figure 2** illustrates different IoU scores and the corresponding membrane segmentation masks for the EcoTest (base kit).

The bounding boxes and segmentation masks of the kit and membrane with the highest detection score are retrieved and a binary segmentation mask is generated for both kit and membrane. Next, the rotation angle is estimated by performing contour detection on the segmentation mask of the kit and membrane, and approximating a minimum-area quadrilateral mask whose corner coordinates can be used to construct a right-angle triangle. In particular, the coordinates of the bottom and top points of the left edge are first obtained, (i.e., (i_{lb}, j_{lb}) and (i_{lt}, j_{lt})), to estimate the angle by $\arctan \frac{j_{lb}-j_{lt}}{i_{lb}-i_{lt}}$. As an alternative, we can also use the right edge to estimate the angle and then use the average of the two estimated angles as the final estimated angle.

The membrane is cropped from the input image with the binary segmentation mask, and is subsequently rotated by the estimated angle. The rotated membrane will have black regions if the estimated angle is greater than zero, and the largest rectangle that doesn't include any black pixels is estimated and extracted as the final membrane to be sent to the classifier. Additionally, we have the capability to compute the homography matrix[20] between the predicted segmentation

mask and bounding box of the kit, and use it to transform the kit of the image to correct for distortion along the pitch axis.

Pre-processing of zone images for classification

As we use the model pre-trained on ImageNet1K as the initialization for our feature extractor, we need to follow the pre-defined image normalization function used in pre-processing ImageNet1K. Specifically, we normalize the pixel values by $i' = {}^{i - mean_c}/_{std_c}$ according to the channel where i is the original pixel value and $(mean_c, std_c)$ varies for different channels. For the channel R, G, and B, the corresponding $(mean_c, std_c)$ are (125.3, 63.0), (123.0, 62.1) and (113.9, 66.7). However, as mentioned before, our approach does not require image calibration and does not perform any other hand-craft pre-processing steps (e.g., white balancing, shadow removal).

Summary of training loss

Mean Squared Error (MSE): Given the predicted image $\widehat{I_e} \in \mathcal{R}^{h \times w}$ and the ground truth edge-enhanced image $I_e \in \mathcal{R}^{h \times w}$ where $(h, w) = (100,64)$ are the height and width of the image. Then, the loss is

$$\mathcal{L}_{MSE}\left(I_e, \widehat{I_e}\right) = \frac{1}{hw} \sum\nolimits_{1 \le i \le h, 1 \le j \le w} \left(I_e(i,j) - \widehat{I_e}(i,j)\right)^2 \tag{1}$$

Cross Entropy Loss (CE): Given the output of the binary classifier $[l_+, l_-]$ and the ground truth class label $y \in \{0,1\}$, the loss function is defined as

$$\mathcal{L}_{CE}([l_+, l_-], y) = y \cdot \log(p_+) + (1 - y)\log(p_-) \tag{2}$$

where $p_+ = \frac{\exp(l_+)}{\exp(l_+)+\exp(l_-)}$ and $p_- = \frac{\exp(l_-)}{\exp(l_+)+\exp(l_-)}$.

Supervised contrastive loss (SupCT) compares a set of features $\boldsymbol{\mathcal{X}} = \{\boldsymbol{x_1}, \boldsymbol{x_2}, \dots, \boldsymbol{x_Q}\} \in \mathcal{R}^{h\times d}$ extracted from the zone images $\{I_\mathbf{1}, I_\mathbf{2}, \dots, I_\boldsymbol{Q}\}$ paired with class labels $\{y_\mathbf{1}, y_\mathbf{2}, \dots, y_\boldsymbol{Q}\}$, where d is the dimension of each feature. Then, for each sample $\boldsymbol{x_i}$, the contrastive loss is defined as

$$\mathcal{L}_{SupCT}(\boldsymbol{x}_i, \boldsymbol{\mathcal{X}}) = \frac{\sum_{j\in\{1,2,\dots,Q\}, j\neq i} \mathbf{1}_{y_i=y_j} \exp \boldsymbol{x}_i \cdot \boldsymbol{x}_j^{\boldsymbol{T}}}{\sum_{j\in\{1,2,\dots,Q\}, j\neq i} \exp \boldsymbol{x}_i \cdot \boldsymbol{x}_j^{\boldsymbol{T}}} \tag{3}$$

where $\mathbf{1}_{y_i=y_j}$ is an indicator function, i.e., 1 if $y_i = y_j$ and 0 otherwise.

We note the parameters within the deep neural network (feature extractor, classifier, and decoder) are not manually set, but updated through gradient backpropagation and chain rule where the gradient is obtained by minimizing the corresponding losses mentioned above. Thus, by varying the hyper-parameters such as training epochs and learning rate listed below, the parameters will be different.

Hyperparameter Selection

Instance segmentation model structure. We used the ResNet50 CNN as the backbone of the Mask R-CNN and pretrained it on the ImageNet1K dataset for model initialization. The backbone has been trained on ImageNet1K as a fully-supervised image classification task among 1,000 classes. We used a hidden layer size of 256 for the mask predictor.

Instance segmentation training. We used 50 epochs and Adam optimizer for all of the training processes. We pretrained the model on a training subset of 50 images of the base kit with a learning rate of 5E-5 and achieved an IoU score of 0.93 on an evaluation set of ten images. We then finetuned the model on the new assay kits with a learning rate of 5E-6 using 10 training images and evaluated the performance on 10 evaluation images. We used the following train-time augmentations: (i) horizontal flip, (ii) scaling, (iii) aspect-ratio modification, (iv) brightness adjustment, (v) contrast adjustment, (vi) hue adjustment, (vii) saturation adjustment, (viii) color distortion, (ix) jitter addition, (x) cropping, (xi) padding, and (xii) Gaussian noise addition. **Supplementary Table 11** shows the results from the test of robustness of the instance segmentation model using bootstrapping.

For training the instance segmentation model on the ABON HIV test we finetuned the pre-trained instance segmentation model on 75 images of the test kit. Hyperparameters used during finetuning are the same as described in the Hyperparameter Selection section. It is important to note that the images from this dataset only featured the membrane as opposed to the entire test kit as in other assay kits. Although no architectural change was needed, minor differences were introduced for the adaptation in the post-processing stage of the instance segmentation model. First, we discarded all model outputs (detection scores, bounding boxes, and segmentation masks) for the kit object class and retained model outputs only for the membrane object class. Consequently, the rotation angle is estimated only on the segmentation mask of the membrane.

Classification model structure. We used the ResNet18 CNN as the feature extractor and pretrained the model on the ImageNet1K dataset for model initialization[21]. The feature extractor

has been trained on ImageNet1K as a fully-supervised image classification task among 1,000 classes. As shown in **Figure 2a**, during the pretraining on base kit images, classifier is configured as a fully connected layer (top output) and the decoder is configured as a stack of three deconvolution layers (bottom output).

Classification model pre-training. Given a training set, all the images were fed into the model in sequence and the loss was calculated for both gradient backpropagation and for updating the model. A single epoch is completed when the model has seen all the images once. 90 epochs were run in our training process. The performance of the model on the validation dataset was determined after each epoch and the model achieving the highest accuracy was selected.

Classification model adaptation & fine-tuning. The network was trained for 100 epochs for each of the new kits with a learning rate of 0.001. Within each epoch, we sample 30 episodes and set Q (number of samples per class) as 32 for each episode. The feature extractor was tuned with a learning rate of 0.0001. Adam optimizer was used for the network parameter update of both the feature extractor and the classifier. The inbuilt PyTorch image transformation functions were used, namely: 1) horizontal flip, 2) Random Rotation, 3) Color Jitter (including grayscale). **Supplementary Table 6** shows the results from the test of robustness of the adapted model on the 4 new test kits using bootstrapping.

Classification model training from scratch. Similar to the initialization step before self-supervision, a ResNet18 CNN is used as the feature extractor which has been trained with the

ImageNet1K dataset as a fully-supervised image classification task. The network is then trained on the training images of the new assay kit with Adam optimizer and a learning rate of 0.001. The same transformation functions used for the adaptation were used here.

Threshold determination and ambiguity region description

We devised an ambiguity region to evaluate the distribution of detection scores (probability of positive class). The ambiguity region is bounded by the detection score thresholds such that an image will be correctly classified only if the probability of the ground truth class is high. The thresholds can be either manually set or statistically estimated with 95% area under the curve. We checked the detection scores of all the images in the evaluation dataset against the ambiguity regions and those images with scores falling in ambiguity region were not classified. We computed the percentage of images that were categorized as ambiguous as well as the accuracy over the images that were classified. Since the detection score for the false predictions were close to 0.5, they fell into the ambiguity region. Therefore, by using this concept of the ambiguity region we were able to treat most of the failure cases as ambiguous while keeping the number of true predictions that fell into the ambiguity region to a minimum. This further increased the classification accuracy among the classified samples consistently over four new target kits.

In general, the thresholds ($\delta_{neg}, \delta_{pos}$) for negative class and positive class were determined individually by feeding the detection score (probability of positive, P_{pos}) of all images of each class into the statistical model and fitting separately. Using the threshold determination of positive class as an example, the steps are explained below: 1) Select

the Inverse Gaussian Distribution as the model template to be fitted[22,23]. The reasons why we select this one-side distribution model are, a) The inverse Gaussian distribution is used to model variables of non-negative values; b) Since the probability output from the model is between 0 and 1, the inverse gaussian distribution is selected as it is tighter within the range [0,1] (i.e., the area under its probability density function (PDF) curve within [0,1] is closer to one), compared to other distribution models such as Gamma distribution which may have an observable tail in [1, infinity) interval. 1) Feed the P_{pos} of all labelled positive zone images into the statistical model and use the fitted parameters to draw the PDF curve. 2) We set the area under the probability distribution curve (between the threshold and the extreme value, i.e., 1 for positive and 0 for negative) as 95% and use Divide and Conquer to find the corresponding threshold value δ, which is threshold for positive class δ_{pos}.

For a negative class, P_{pos} is still used as input to find the classification score threshold δ_{neg}. For the convenience of presentation, $[\delta_{neg}, \delta_{pos}]$ is used to denote the ambiguity region where images with $\delta_{neg} \leq P_{pos} \leq \delta_{pos}$ will not be classified since they fall within the region, and the images with $P_{pos} \leq \delta_{neg}$ or $P_{pos} \geq \delta_{pos}$ are classified as negative or positive respectively. The ratio of the unclassified images with respect to the entire evaluation set is reported as the percentage of ambiguous cases.

Calculation of execution time

For the drive-through study and in general, the overall execution time was measured from sending the AWS S3 pathname of the test kit image to the Lambda function for processing, to the

return of the test assessment classification results by the function. The specific processing steps were: 1) Image down sampling to 800 px height (Inline python code), 2) Object detector (AWS SageMaker Real-Time Inference), 3) Process object masks, rotate to vertical, compute object overlap, apply homography correction, extract full resolution membrane image for classification (Inline python code), 4) Classifier (AWS SageMaker Real-Time Inference), 5) Assemble and return JSON results data (Inline python code). For the object detector model, an NVIDIA T4 Tensor Core (16 GB GPU memory) instance was employed. The classifier model employed a CPU instance.

D. Supplementary References

1 Joung, H.-A. *et al.* Point-of-care serodiagnostic test for early-stage lyme disease using a multiplexed paper-based immunoassay and machine learning. *Acs Nano* **14**, 229-240 (2019).

2 Shokr, A. *et al.* Mobile Health (mHealth) Viral Diagnostics Enabled with Adaptive Adversarial Learning. *Acs Nano* **15**, 665-673, doi:10.1021/acsnano.0c06807 (2021).

3 Turbé, V. *et al.* Deep learning of HIV field-based rapid tests. *Nature medicine*, 1-6 (2021).

4 Wong, N. C. K. *et al.* Machine learning to support visual auditing of home-based lateral flow immunoassay self-test results for SARS-CoV-2 antibodies. *Communications Medicine* **2**, doi:10.1038/s43856-022-00146-z (2022).

5 Mendels, D.-A. *et al.* Using artificial intelligence to improve COVID-19 rapid diagnostic test result interpretation. *Proceedings of the National Academy of Sciences* **118** (2021).

6 Carrio, A., Sampedro, C., Sanchez-Lopez, J. L., Pimienta, M. & Campoy, P. Automated low-cost smartphone-based lateral flow saliva test reader for drugs-of-abuse detection. *Sensors* **15**, 29569-29593 (2015).

7 Ballard, Z. S. *et al.* Deep learning-enabled point-of-care sensing using multiplexed paper-based sensors. *NPJ Digit Med* **3**, 1-8 (2020).

8 Li, J. *et al.* Development of a smartphone-based quantum dot lateral flow immunoassay strip for ultrasensitive detection of anti-SARS-CoV-2 IgG and neutralizing antibodies. *International Journal of Infectious Diseases* **121**, 58-65, doi:10.1016/j.ijid.2022.04.042 (2022).

9 Dell, N. & Borriello, G. in *Proceedings of the 3rd ACM Symposium on Computing for Development.* 1-10.

10 Ruppert, C., Phogat, N., Laufer, S., Kohl, M. & Deigner, H.-P. A smartphone readout system for gold nanoparticle-based lateral flow assays: application to monitoring of digoxigenin. *Microchimica Acta* **186**, 119 (2019).

11 Wang, W., Stafford, J. W., Banik, D. & Keller, M. D. in *Optics and Biophotonics in Low-Resource Settings VIII.* 69-76 (SPIE).

12 Balán, I. C. *et al.* Acceptability and Use of a Dual HIV/Syphilis Rapid Test and Accompanying Smartphone App to Facilitate Self-and Partner-Testing Among Cisgender Men and Transgender Women Who Have Sex with Men. *AIDS and Behavior*, 1-12 (2021).

13 Balán, I. C. *et al.* SMARTtest: A Smartphone App to Facilitate HIV and Syphilis Self- and Partner-Testing, Interpretation of Results, and Linkage to Care. *AIDS and Behavior*, 1-14 (2019).

14 Lee, S. *et al.* Machine-learning-assisted lateral flow assay for COVID-19 and influenza detection. *Available at SSRN 4073623* (2022).

15 Beggs, A. D. *et al.* Machine learning for determining lateral flow device results for testing of SARS-CoV-2 infection in asymptomatic populations. *Cell Reports Medicine* **3**, 100784, doi:10.1016/j.xcrm.2022.100784 (2022).

16 He, K., Gkioxari, G., Dollar, P. & Girshick, R. Mask R-CNN. *IEEE Trans Pattern Anal Mach Intell* **42**, 386-397, doi:10.1109/TPAMI.2018.2844175 (2020).

17 Ren, S., He, K., Girshick, R. & Sun, J. Faster R-CNN: Towards Real-Time Object Detection with Region Proposal Networks. *IEEE Trans Pattern Anal Mach Intell* **39**, 1137-1149, doi:10.1109/TPAMI.2016.2577031 (2017).

18 Girshick, R. in *Proceedings of the IEEE international conference on computer vision.* 1440-1448.

19 Minaee, S. *et al.* Image segmentation using deep learning: A survey. *IEEE Transactions on Pattern Analysis Machine Intelligence* (2021).

20 Geetha Kiran, A. & Murali, S. Automatic rectification of perspective distortion from a single image using plane homography. *J. Comput. Sci. Appl* **3**, 47-58 (2013).

21 Deng, J. *et al.* in *2009 IEEE conference on computer vision and pattern recognition.* 248-255 (Ieee).

22 Folks, J. L. & Chhikara, R. S. The inverse Gaussian distribution and its statistical application—a review. *Journal of the Royal Statistical Society: Series B* **40**, 263-275 (1978).

23 Chhikara, R. *The inverse Gaussian distribution: theory: methodology, and applications*. Vol. 95 (CRC Press, 1988).
