# Supplementary material for: Rapidly adaptable automated interpretation of point-of-care COVID-19 diagnostics
Source: Commun Med (Lond). 2023 Jun 23;3:91. doi: 10.1038/s43856-023-00312-x (PMC10290128; doi:10.1038/s43856-023-00312-x)
Supplement: Supplementary file 5 — Reporting Summary [file 43856_2023_312_MOESM5_ESM.pdf]

nature portfolio

Corresponding author(s): Samuel K. Sia

Last updated by author(s): May 15, 2023

Reporting Summary

Nature Portfolio wishes to improve the reproducibility of the work that we publish. This form provides structure for consistency and transparency in reporting. For further information on Nature Portfolio policies, see our Editorial Policies and the Editorial Policy Checklist.

Statistics

For all statistical analyses, confirm that the following items are present in the figure legend, table legend, main text, or Methods section.

n/a	Confirmed	
☐	☒	The exact sample size (*n*) for each experimental group/condition, given as a discrete number and unit of measurement
☐	☒	A statement on whether measurements were taken from distinct samples or whether the same sample was measured repeatedly
☒	☐	The statistical test(s) used AND whether they are one- or two-sided<br>*Only common tests should be described solely by name; describe more complex techniques in the Methods section.*
☒	☐	A description of all covariates tested
☒	☐	A description of any assumptions or corrections, such as tests of normality and adjustment for multiple comparisons
☒	☐	A full description of the statistical parameters including central tendency (e.g. means) or other basic estimates (e.g. regression coefficient) AND variation (e.g. standard deviation) or associated estimates of uncertainty (e.g. confidence intervals)
☒	☐	For null hypothesis testing, the test statistic (e.g. *F*, *t*, *r*) with confidence intervals, effect sizes, degrees of freedom and *P* value noted<br>*Give P values as exact values whenever suitable.*
☒	☐	For Bayesian analysis, information on the choice of priors and Markov chain Monte Carlo settings
☒	☐	For hierarchical and complex designs, identification of the appropriate level for tests and full reporting of outcomes
☒	☐	Estimates of effect sizes (e.g. Cohen's *d*, Pearson's *r*), indicating how they were calculated

Our web collection on statistics for biologists contains articles on many of the points above.

Software and code

Policy information about availability of computer code

Data collection	As specified in the manuscript, test kit images were collected using an iPhone X.
Data analysis	The custom algorithm used to interpret test kit images is available to download from github. Data analysis for data presented in figures was conducted using Microsoft Excel and Python.

For manuscripts utilizing custom algorithms or software that are central to the research but not yet described in published literature, software must be made available to editors and reviewers. We strongly encourage code deposition in a community repository (e.g. GitHub). See the Nature Portfolio guidelines for submitting code & software for further information.

Data

Policy information about availability of data

All manuscripts must include a data availability statement. This statement should provide the following information, where applicable:

- Accession codes, unique identifiers, or web links for publicly available datasets
- A description of any restrictions on data availability
- For clinical datasets or third party data, please ensure that the statement adheres to our policy

All test kits images used for base model training, adaptation, and for the drive through study are available to download from the following link upon request.
https://drive.google.com/drive/folders/1PJRmiCviniQShOJcBxmDFpOCkIscRGZ9?usp=sharing

March 2021

The dataset used for evaluating the algorithm on an HIV rapid test kits is available here: https://data.ahri.org/index.php/catalog/923.8

Source data for Fig 4. and Figure 7C. is available in Supplementary Data 1 and 2 respectively.

All other data is available from the corresponding author on reasonable request.

Human research participants

Policy information about studies involving human research participants and Sex and Gender in Research.

Reporting on sex and gender	N/A
Population characteristics	N/A
Recruitment	Participants in the antigen testing arm were recruited via the drive-through COVID-19 testing site at an academic hospital campus (IRB protocol 20-010688).Participants in the antibody testing arm were contacted 2 weeks after a positive PCR result and invited to return ≥ 3 weeks from their positive PCR result and ≥ 2 weeks from symptom resolution (IRB protocol 20-004544). For the comparative assessment study, participants were recruited by word of mouth using signs posted outside Mayo Clinic at Arizona State University Health Futures Center.
Ethics oversight	Mayo Clinic

Note that full information on the approval of the study protocol must also be provided in the manuscript.

Field-specific reporting

Please select the one below that is the best fit for your research. If you are not sure, read the appropriate sections before making your selection.

☒ Life sciences ☐ Behavioural & social sciences ☐ Ecological, evolutionary & environmental sciences

For a reference copy of the document with all sections, see nature.com/documents/nr-reporting-summary-flat.pdf

Life sciences study design

All studies must disclose on these points even when the disclosure is negative.

Sample size	For the drive-through study, the sample size was determined by the number of people who consented at the drive-through clinic. For the comparative assessment, 25 participants were recruited as a proof of concept study.
Data exclusions	In the comparative assessment study, two participants were excluded from analysis due to failure to meet the English-speaking criteria. For both studies, any test kit that produced an invalid test-kit result (i.e., no control band present) was excluded from analysis.
Replication	In the drive-through study, all participants were given a tray with the required components to conduct a rapid test and capture an image (Figure 5A). There were asked to stay in their vehicle to simulate a home environment. For the comparative assessment study, all test kits were ran with specific virus titers (Supplementary Table 10) by study staff.
Randomization	For the drive-through study, the antigen testing arm was recruited at a drive-through testing site for PCR testing. The antibody testing arm was recruited 2 weeks following a positive PCR result from the drive-through clinic.
Blinding	In both studies, the interpretation of the algorithm was not sent to the user. In the comparative assessment study, the expert interpreter was not aware of the virus titers used for each rapid test.

Reporting for specific materials, systems and methods

We require information from authors about some types of materials, experimental systems and methods used in many studies. Here, indicate whether each material, system or method listed is relevant to your study. If you are not sure if a list item applies to your research, read the appropriate section before selecting a response.

Materials & experimental systems

n/a	Involved in the study
☒	☐ Antibodies
☒	☐ Eukaryotic cell lines
☒	☐ Palaeontology and archaeology
☒	☐ Animals and other organisms
☒	☐ Clinical data
☒	☐ Dual use research of concern

Methods

n/a	Involved in the study
☒	☐ ChIP-seq
☒	☐ Flow cytometry
☒	☐ MRI-based neuroimaging
