# Supplementary material for: Rapidly adaptable automated interpretation of point-of-care COVID-19 diagnostics
Source: Commun Med (Lond). 2023 Jun 23;3:91. doi: 10.1038/s43856-023-00312-x (PMC10290128; doi:10.1038/s43856-023-00312-x)
Supplement: Supplementary file 6 — Description of Additional Supplementary Files [file 43856_2023_312_MOESM6_ESM.pdf]

Description of Additional Supplementary File

File Name: Supplementary Data 1

Description: Source data for Figure 4

File Name: Supplementary Data 2

Description: Source data for Figure 7C

File Name: Supplementary Video 1

Description: Overview of sample dashboard for real-time population disease surveillance and visualization of data generated by the algorithm of this study as collected from smartphones
